# Outcomes of a 3-day transparent film dressing protocol after hypospadias repair

**DOI:** 10.1038/s41598-024-68059-6

**Published:** 2024-10-15

**Authors:** Vita Indriasari, Yodya Evila, Rizki Diposarosa, Yoni F. Syukriani, Dedi Rachmadi

**Affiliations:** 1 Pediatric Surgery Division, Department of Surgery, Faculty of Medicine, Universitas Padjadjaran/Dr. Hasan Sadikin General Hospital, Bandung, Indonesia; 2 Department of Forensic & Legal Medicine, Faculty of Medicine, Universitas Padjadjaran/Dr. Hasan Sadikin General Hospital, Bandung, Indonesia; 3 Division of Nephrology, Department of Pediatrics, Faculty of Medicine, Universitas Padjadjaran/Dr. Hasan Sadikin General Hospital, Bandung, Indonesia

**Keywords:** Hypospadias, Surgical site infection, Transparent film dressing, Medical research, Urology, Paediatric urology, Urogenital diseases

## Abstract

This study aimed to evaluate a 3-day transparent film dressing protocol after hypospadias repair. A retrospective observational study was conducted in boys with hypospadias who were operated in our institution between 2022 and 2023. Postoperatively, the penis was wrapped with a transparent film dressing, which was removed after 3 days. Postoperative complications were observed until postoperative day 14. The associations of age, meatal location, and type of procedure were analyzed using Chi square, Fisher exact, Mann Whitney, and Kruskall Wallis test (p < 0.05 = significant). Sixty-five patients were studied. Median age was five years, the majority had proximal meatus (58.5%), and underwent urethroplasty (76.9%). After dressing removal, positive bacterial culture was found in 43.1%, mild penile edema in 33.8%, bleeding in 10.8%, and SSI in 49.2% of cases, with pus formation (10.8%), dehiscence (9.2%), and urethrocutaneous fistula (10% after urethroplasty procedure). Surgical site infection and positive culture were significantly higher in patients with proximal meatus compared to distal (p = 0.031, p = 0.019; respectively). A 3-day transparent film dressing prevented penile edema and bleeding in most cases. However, the rate of SSI and positive wound culture was high, and was associated with proximal meatal location.

## Introduction

Surgical techniques for hypospadias have undergone continuous evolution to improve postoperative outcomes^1^. However, its postoperative management including selection of penile dressing remains challenging. Various dressings, regarding the type of material, method, and duration used, were described, ranging from a totally concealing dressing to a partially, or completely undressed wound^2^. However, the ideal one for hypospadias remains debatable.

Currently, many surgeons use simple partially concealing dressing after hypospadias repair^3–9^. One of which is a partially concealing transparent film such as Tegaderm^®^ because it is simple, cost-effective, sterile, waterproof, and allow easy visualization for recognizing early complications^8,10^ . In 1996, Duckett recommended to use it for 48–72 h depending on the type of hypospadias surgery performed. Other studies reported by Retik et al. and Snodgrass et al. also used a transparent film dressing to allow easier observation with excellent functions^11,12^.

The main purpose of a proper penile dressing after hypospadias repair is to provide immobilization for prevention of hematoma and edema^13^, however, potential problems may still occur. Dressing may decrease blood flow to the penis, which is important for healing. Contamination may cause infection and lead to development of urethrocutaneous fistula^14^. At our institution, due to its practicality, partially concealed transparent film dressing was used for 3 days following hypospadias repair. However, detailed postoperative wound condition after dressing removal have not been evaluated. The aim of this study was to evaluate the use of a 3-day transparent film dressing protocol for hypospadias repair over two years at a tertiary referral hospital.

## Methods

A retrospective observational study was conducted on boys with hypospadias between 2022 and 2023. The inclusion criteria were all children aged up to 18 years old, diagnosed with hypospadias and underwent surgical repair. The exclusion criteria are patients with urethral meatus located on the ventral side of the penis due to acquired conditions such as trauma or iatrogenic, patients with hematologic disease, or patients with immune disorders. This study has been approved by the Research Ethics Committee of Dr. Hasan Sadikin General Hospital (No. LB.02.01/X.6.5/126/2022), and the study was performed in accordance with relevant guidelines. Informed consent regarding the preoperative procedure including post operative wound care and dressing methods applied to all hypospadias patients who underwent surgery, was obtained from all patients and/or their legal guardians.

Preoperatively, penile biometry was assessed, including the location of the urethral meatus. Urethral meatus at the glans, corona, distal, and midshaft were considered distal location, whereas the proximal shaft, penoscrotal, scrotal, and perineal meatal locations were considered proximal. Genital skin preparation was performed the night before surgery using chlorhexidine as an antiseptic solution. All patients were operated under general anesthesia. We did one stage repair for distal hypospadias with less than 30-degree penile curvature using tubularized incised plate (TIP) technique. For proximal hypospadias, we performed staged repair. At the first stage we performed penile straightening by degloving of penis, with or without dividing the urethral plate, or mobilizing the spongious body or ventral corpotomy (depended on the depth/location of the curvature). In this stage, scrotoplasty might be done if there is penoscrotal transposition. The following step was urethroplasty using periurethral plate skin flap forming a tubularized urethra. Dartos fascia or tunica vaginalis was used for secondary layer. Urethroplasty could be done completely until the tip of glans or staged, depending on the meatal location. We used a feeding tube sized 8–10 Fr as urethral stent and bladder drainage, depending on the size of the neourethra. The procedures were done by 3 pediatric surgeons, all are specialized in pediatric urogenital surgery.

Postoperatively, whether after one stage or subsequent stage of hypospadias repair, all patients were received the same penile dressing protocol. The penis was wrapped with two layers of transparent film dressing (Tegaderm^®^) circumferentially, from the penoscrotal junction to the glans, excluding the urethral meatus. Tegaderm^®^ composition consisted of 60–70% hydrocolloid adhesive. The dressing was removed after 3 days. Immediately after removal and, skin swab and microorganism culture were performed. Wound was evaluated until 14 days postsurgery. The incidence of penile edema, bleeding, SSI, pus formation, wound dehiscence, positive wound culture, and urethrocutaneous fistula after urethroplasty was recorded. SSI criteria:

1. Infections that occur within 30 days after surgery and
2. One of the conditions below:
  1. Purulent drainage from the incision
  2. Causative organisms can be isolated from tissue cultures taken aseptically
  3. At least there are:
    1 as follows: pain, localized swelling, redness, or warmth to the touch, and
    Diagnosis of SSI by the surgeon

The associations between age at surgery, meatal location, type of procedure and the occurrence of postoperative complications after dressing removal were analyzed using the chi-square test and Fisher’s exact test for categorical variables; and the Mann–Whitney and Kruskall-Wallis tests for numeric variables (p < 0.05 was considered significant).

### Ethics approval

Ethical approval was waived by the Research Ethics Committee of Dr. Hasan Sadikin General Hospital No. LB.02.01/X.6.5/126/2022 in view of the retrospective nature of the study and all the procedures being performed were part of the routine care.

## Results

During the study period, 65 hypospadias patients were treated in our institution. The characteristics of the patients and postoperative complications are described in Table 1. Of all patients who had SSI, 87.5% had positive culture result with majority of Gram-negative bacteria (75% of all patients with positive culture result). The most common organisms were *Staphylococcus* spp. (32%), followed by *Escherichia* spp. (25%), and *Enterococcus faecalis* (21,4%). *Enterobacter* spp. (10,7%), *Acinetobacter baumanii* (10,7%), *Klebsiella aerogenes* (7,1%), *Pantoea aeglomerans* (3,5%), *Pseudomonas putida* (3,5%), *Streptococcus mitis* (3,5%), and *Candida tropicalis* (3,5%) were also found.

**Table 1:** Characteristics of hypospadias patients.

Variables	n = 65
Age (years)	
Median (IQR)	5 (2.8–8.3)
Min–max	0.5–18.0
Meatal location, n (%)	
Proximal	38 (58.5)
Distal	27 (41.5)
Type of procedure, n (%)	
Urethroplasty	50 (76.9)
Chordectomy	15 (23.1)
Postoperative complications, n (%)	
Edema	22 (33.8)
Bleeding	7 (10.8)
SSI	32 (49.2)
Pus formation	7 (10.8)
Wound dehiscence	6 (9.2)
Urethrocutaneous fistula	5 (7.7)
Positive bacterial culture	28 (43.1)
Negative bacterial culture	4 (6.1)
SSI depth	4 (6.1)
Deep	6 (9.2)
Superficial	26 (40.0)

*n* frequency, *%* percentage, *IQR* inter quartile range.

**Figure 1:**
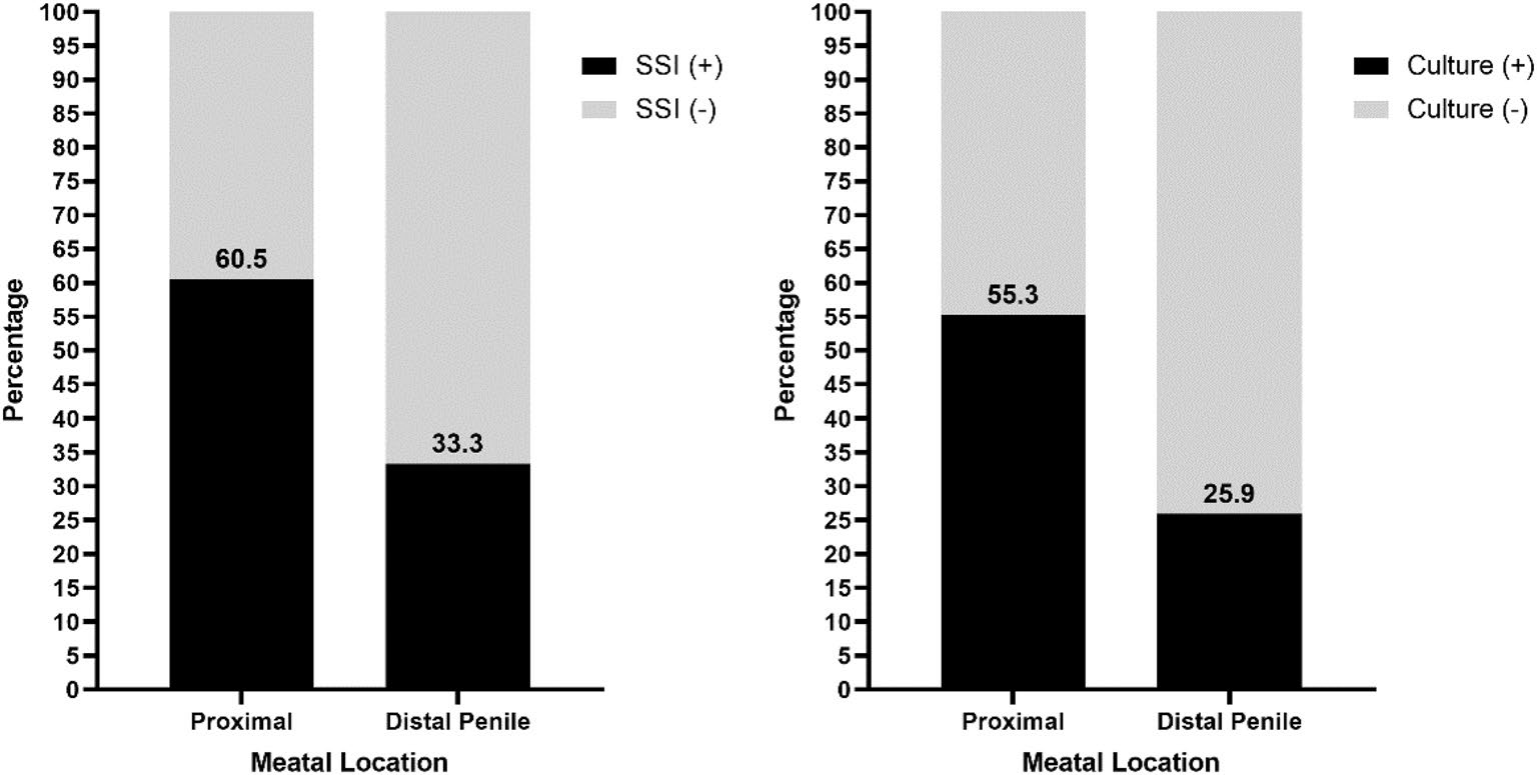
Barchart association of meatal location and incidence of SSI and bacterial culture after dressing removal in hypospadias patients.

We analyzed factors that may associated with postoperative complications in our patients. Median age was higher in patients who developed pus at the wound compared to those without pus, however it was not significantly different (p = 0.641) (Table 2). The proportion of patients with SSI was significantly higher in proximal meatus group compared to the distal one (p = 0.031) (Table 3, Fig. 1). Positive wound culture was also more common in patients with proximal meatus There was a tendency for positive sign of SSI with positive culture to be higher in patients with proximal meatus (55.3%) compared to distal (25.9%) with a p value of 0.062. There was no significant difference in the distribution of postoperative complications after removal of dressing between patients who underwent urethroplasty and chordectomy (p > 0.05) (Table 4) (Supplementary Information).

**Table 2:** Association of age and postoperative complication after dressing removal in hypospadias patients.

Variable	Age (year)	p value
	Median (IQR)	
Postoperative complications n (%)		
Edema		0.076^a^
Yes	4.7 (2.0–10.0)	
No	5.5 (3.0–8.3)	
Bleeding		0.379^a^
Yes	7.0 (5.0–10.0)	
No	5.0 (2.4–8.7)	
SSI		0.494^a^
Yes	5.2 (2.0–10.0)	
No	5.0 (4.0–7.9)	
Pus formation		0.641^a^
Yes	12.9 (1.0–14.0)	
No	5.0 (2.7–8.0)	
Dehiscence		0.525^a^
Yes	6.0 (1.4–11.7)	
No	5.0 (2.9–8.0)	
Urethrocutaneous fistula		0.246^a^
Yes	4.0 (2.1–12.2)	
No	5.1 (2.7–9.6)	
Positive bacterial culture		0.569^a^
Yes	5.2 (2.0–10.0)	
No	5.0 (4.0–8.0)	
SSI depth		0.525^b^
Deep	6.9 (5,2–14.0)	
Superficial	5.0 (2.45–10.0)	
Normal (no SSI)	5.0 (2.0–8.0)	
SSI signs and bacterial culture		0.395^b^
Signs + dan Culture +	5.25 (2.0–10.0)	
Signs + dan Culture –	6.0 (1.6–15.2)	
Signs – dan Culture –	5.0 (4.0–7.9)	

p value using ^a^Mann Whitney test, ^b^Kruskall Wallis test, significant p < 0.05.

**Table 3:** Association of meatal location and postoperative complication after dressing removal in hypospadias patients.

Variables	Meatal Location		p value
	Proximal n = 38	Distal n = 27	
Postoperative complications n (%)			
Edema	14 (36.8)	8 (29.6)	0.545^a^
Bleeding	4 (10.5)	3 (11.1)	1.000^b^
SSI	23 (60.5)	9 (33.3)	**0.031**^a^*
Pus formation	5 (13.2)	2 (7.4)	0.690^b^
Wound dehiscence	4 (10.5)	2 (7.4)	1.000^b^
Urethrocutaneous fistula	3 (7.9)	2 (7.4)	1.000^b^
Positive bacterial culture	21 (55.3)	7 (25.9)	**0.019**^a^*
SSI depth			0.093^a^
Deep	4 (10.5)	2 (7.4)	
Superficial	19 (50.0)	7 (25.9)	
Normal (no SSI)	15 (39.5)	18 (66.7)	
SSI signs and bacterial culture			0.062^a^
Signs + dan culture +	21 (55.3)	7 (25.9)	
Signs + dan culture –	2 (5.3)	2 (7.4)	
Signs – dan culture –	15 (39.4)	18 (66.7)	

p value using ^a^Chi square test, ^b^Fisher Exact test, *significant p < 0.05.

Significant values are in bold.

**Table 4:** Association of type of procedure and postoperative complication after compressive dressing’s removal in patients with hypospadias.

Variables	Type of Procedure		p value
	Urethroplasty n = 50	Chordectomy n = 15	
Postoperative complications n (%)			
Edema	17 (34.0)	5 (33.3)	0.962^a^
Bleeding	5 (10.0)	2 (13.3)	0.658^b^
SSI	23 (46.0)	9 (60.0)	0.341^a^
Pus formation	6 (12.0)	1 (6.7)	1.000^b^
Wound dehiscence	5 (10.0)	1 (6.7)	1.000^b^
Positive bacterial culture	19 (38.0)	9 (60.0)	0.131^a^
SSI depth			0.484^a^
Deep	5 (10.0)	1 (6.7)	
Superficial	18 (36.0)	8 (53.3)	
Normal (no SSI)	27 (54.0)	6 (40.0)	
SSI signs and bacterial culture			0.230^a^
Signs + dan Culture +	19 (38.0)	9 (60.0)	
Signs + dan Culture –	4 (8.0)	0 (0.0)	
Signs – dan Culture –	27 (54.0)	6 (40.0)	

p value using ^a^Chi Square test, ^b^Fisher Exact test, significant p < 0.05.

## Discussion

To our knowledge this is one of few studies that focused on detailed early postoperative wound evaluation after transparent film dressing removal in hypospadias patients. A retrospective cohort study comparing the outcomes of 3-day hydrocolloid dressing and petroleum-impregnated gauze showed no significant difference in postoperative outcomes such as fistula, stenosis, wound dehiscence, or chordee^15^. Another study showed that there was no significant different in the incidence of meatal regression, fistula, stenosis, chordee, cosmesis, and stent migration between hypospadias patients who applied transparent dressing for 2 days and those without dressing^12^. However, those studies did not evaluate early postoperative outcomes especially the clinical signs of SSI including bacterial culture on the wound after dressing removal. An earlier cohort study comparing three dressing methods: compressive dressing, transparent bio membrane dressing, and no dressing at all, also did not show significant difference in outcomes, in which the SSI was not evaluated in detail. In transparent dressing group, the duration of dressing was variable (1 to > 8 days), but the outcome of different dressing durations was not compared^16^.

We found a 33.8% incidence of edema after dressing removal, which was higher than other reported studies^17^. However, all the symptoms were mild and spontaneously resolved without further treatment. Wide dissection of penile tissue and degloving of the penis may cause postoperative edema accompanied with accumulation of fluid that inhibits proper fusion of the tissue layer and predisposes patients to infections and poor wound healing^16,17^. Compressive dressings play a significant role in restricting edema and supporting the penis while the patient is in an upright position; therefore, they enhance lymph drainage^18^. Edema may be aggravated following the removal of compressive dressing. Surgeons usually concerned about edema because it may cut through repair and stitches. However, in this study all the cases of wound dehiscence (9,2%) were due to SSI. While many surgeons assumed that dressing can limits the degree of postoperative edema, McLorie et al. and Karakaya et. al reported no differences in the degree of edema between hypospadias patients treated with and without dressing^11,16,19^. Earlier study found that silicone foam elastomer can be an excellent choice of hypospadias dressing given the advantage that it is nonadherent and compressive enough to avoid swelling, but the dressing could not allow visualization of the penis and more expensive compared to the transparent film dressing we use^5^. Other study comparing conventional dressing (pressure wrap dressing) and cyanoacrylate (CA) glue showed that CA glue is a good alternative dressing that could prevent urine and stool contamination and prevent edema and hematoma, however it is not easily accessible and increase cost for patients^20^.

Postoperative bleeding was also evaluated in this study. The exact incidences of bleeding and hematoma after hypospadias repair have not been reported in the literature. The usual cause of bleeding is from resected corpus spongiosum during chordectomy or inadequate hemostasis. Bleeding can be minimized by proper plane of dissection and meticulous hemostasis and compressive dressing applied postoperatively^17,18^. Dressing provides gentle compression for hemostasis and prevents hematoma that could disturb the blood supply to healing tissue^11,19^. In this study, bleeding was observed in 10,8% of cases but the bleeding was minor and stopped spontaneously.

Despite the preoperative skin preparation, positive bacterial cultures on the wound were found in 43,1% of cases immediately after dressing removal, even before the patients’ exhibited signs of SSI. Dressing is likely to become wet from skin sweating, especially impermeable one, as used in this study. The material may create a suitable environment for the colonization of microorganisms. If a membrane dressing is not attached well to the skin, a gap may form, and bacterial contamination may be trapped underneath the film. It may be from urine dripping through the urethra and soiling following bowel movement^11,12,21^. The most common pathogen found in this study was gram-negative bacteria, similar to the findings of previous studies performed by Ratan et al. and Sanders et al.^21,22^. Normally, the skin is colonized by several commensal bacteria, but the site of surgery, body temperature, and personal hygiene may influence surgical wound contamination and alter the composition of the body flora. It is generally assumed that the perineum is often heavily contaminated with microorganisms originating from the anus^21,22^.

In this study, signs of SSI such as hyperaemia and pus formation, occurred on postoperative days five to seven. However, evaluating early signs of SSI after hypospadias repair in young children is quite challenging. The penile is relatively small, so that localized swelling and pain caused by SSI are somewhat difficult to differentiate from penile edema due to surgical dissection. Previously, a diagnosis of SSI was made after hyperaemia and pus formation became evident on the wound. However, in this study, immediately after dressing removal (on postoperative day three), 43.1% of the patients had a positive wound culture, which fulfilled the criteria of SSI, while pus developed later in only 10.8% of the cases. This is an indication that more cases were actually at risk for developing further complications than we previously thought. However, despite a high rate of SSI, wound dehiscence developed in only 9.2% of patients, and urethrocutaneous fistula developed in 10% of urethroplasty patients.

Impaired penile circulation and colonization by microorganisms after compressive dressing are thought to influence the development of SSI^11,12,18^. The duration of dressing may also contribute for the high rate of SSI in this study. Although dressings applied to closed wounds should be removed on the third or fourth postoperative day^23^, microscopic contamination may have already occurred in it. Murakami et al. reported that removing dressings one day after surgery effectively prevented infection and decreased the number of pathogenic floras^24^. Other study by Roberto et al. evaluated foam dressing that is removed after 5–10 days, the dressing, being non-adherent in the wound area, is observed to be easily peeled off with no need of analgesics and minimal discomfort to the child but we thought delayed dressing removal could lengthen the duration of hospital stay. Another study regarding dressing removal in hypospadias by McLorie et al. describes longer duration of dressing could cause odor and skin irritation^16^.

We also analyzed other factors that may be associated with postoperative complications. The median age of our patients was five years old, which is relatively older than the current recommendation age for hypospadias repair. Current practice recommends surgery performed between six and 18 months of age considering anesthetic risks and age-dependent tissue dimensions^2,25^. Several studies suggest that initial hypospadias repair at an older age could be associated with more postoperative complications, which is consistent with the findings of our study^26–28^. Factors such as erections during the postoperative period might cause hematoma and wound breakdown. In postpubertal males, the amount of urethral secretions produced may provide an environment that also promotes infections^26,28^. Another study involving hypospadias repair by Hensle et al. noted a 52.3% complication rate for hypospadias repair in 42 adults^29^. Marocco et al. reported an increased number of wound-related complications in patients older than 1 year (18.7%)^28^. Dodson et al. reported short-term and long-term complications in 15 of 31 patients aged 10 years or older who underwent hypospadias^26^. In this study, patients who developed pus were older than patients without pus. However, the difference was not significant.

In this study, we found a relationship between meatal location and the incidence of SSI (p = 0.031) and positive culture (p = 0.019) in hypospadias patients who applied this dressing protocol. The proportion of patients who developed SSIs and had positive cultures was greater in the proximal meatus group. The proximity of the urethral meatus to the anus increases the number of contaminations by feces and the humidity of the wound environment^30^.

The location of the meatus is an important variable in determining the surgical technique used. With more proximally located meatus and severe degree hypospadias, the technique used could be more complex and some patients might need staged surgery^31,32^.

While it prevented postoperative edema and bleeding in most cases, high rate of SSI and positive wound culture in this study were a concern. Although this was not followed by a high incidence of wound dehiscence and fistula, perioperative infection prevention and control should be improved. The need for postoperative dressing should also be reviewed, and if they are still needed, further study should be conducted to compare dressing types, materials, and methods of monitoring. This study has several limitations. Our subjects were of varying age groups with different type of hypospadias and procedures that may have interfered with the outcome of the dressing protocol. Additionally, the outcomes of this protocol and other dressing methods were not compared.

## Conclusion

A 3-day transparent film dressing prevented penile edema and bleeding in most cases. However, the rate of SSI and positive wound culture was high, and was associated with proximal meatal location.

## Supplementary Information

Supplementary Information 1.

Supplementary Information 2.

## Data Availability

The datasets used and/or analysed during the current study available from the corresponding author on reasonable request.
