# Supplementary material for: Outcomes of a 3-day transparent film dressing protocol after hypospadias repair
Source: Sci Rep. 2024 Oct 15;14:24192. doi: 10.1038/s41598-024-68059-6 (PMC11480378; doi:10.1038/s41598-024-68059-6)
Supplement: Supplementary file 2 — Supplementary Information 2. [file 41598_2024_68059_MOESM2_ESM.docx]

**Frequencies**

**Frequency Table**

| **Urethral Meatus** | | | | | |
| --- | --- | --- | --- | --- | --- |
|  | | Frequency | Percent | Valid Percent | Cumulative Percent |
| Valid | Proximal | 38 | 58.5 | 58.5 | 58.5 |
|  | Distal | 27 | 41.5 | 41.5 | 100.0 |
|  | Total | 65 | 100.0 | 100.0 |  |

| **Treatment** | | | | | |
| --- | --- | --- | --- | --- | --- |
|  | | Frequency | Percent | Valid Percent | Cumulative Percent |
| Valid | Urethroplasty | 50 | 76.9 | 76.9 | 76.9 |
|  | Chordectomy | 15 | 23.1 | 23.1 | 100.0 |
|  | Total | 65 | 100.0 | 100.0 |  |

| **edema** | | | | | |
| --- | --- | --- | --- | --- | --- |
|  | | Frequency | Percent | Valid Percent | Cumulative Percent |
| Valid | No | 43 | 66.2 | 66.2 | 66.2 |
|  | Yes | 22 | 33.8 | 33.8 | 100.0 |
|  | Total | 65 | 100.0 | 100.0 |  |

| **Bleeding** | | | | | |
| --- | --- | --- | --- | --- | --- |
|  | | Frequency | Percent | Valid Percent | Cumulative Percent |
| Valid | No | 58 | 89.2 | 89.2 | 89.2 |
|  | Yes | 7 | 10.8 | 10.8 | 100.0 |
|  | Total | 65 | 100.0 | 100.0 |  |

| **Pus** | | | | | |
| --- | --- | --- | --- | --- | --- |
|  | | Frequency | Percent | Valid Percent | Cumulative Percent |
| Valid | No | 58 | 89.2 | 89.2 | 89.2 |
|  | Yes | 7 | 10.8 | 10.8 | 100.0 |
|  | Total | 65 | 100.0 | 100.0 |  |

| **Dehiscence** | | | | | |
| --- | --- | --- | --- | --- | --- |
|  | | Frequency | Percent | Valid Percent | Cumulative Percent |
| Valid | No | 59 | 90.8 | 90.8 | 90.8 |
|  | Yes | 6 | 9.2 | 9.2 | 100.0 |
|  | Total | 65 | 100.0 | 100.0 |  |

| **Fistula** | | | | | |
| --- | --- | --- | --- | --- | --- |
|  | | Frequency | Percent | Valid Percent | Cumulative Percent |
| Valid | No | 60 | 92.3 | 92.3 | 92.3 |
|  | Yes | 5 | 7.7 | 7.7 | 100.0 |
|  | Total | 65 | 100.0 | 100.0 |  |

| **SSI Clinically +** | | | | | |
| --- | --- | --- | --- | --- | --- |
|  | | Frequency | Percent | Valid Percent | Cumulative Percent |
| Valid | No. | 33 | 50.8 | 50.8 | 50.8 |
|  | Yes | 32 | 49.2 | 49.2 | 100.0 |
|  | Total | 65 | 100.0 | 100.0 |  |

| **Positive culture** | | | | | |
| --- | --- | --- | --- | --- | --- |
|  | | Frequency | Percent | Valid Percent | Cumulative Percent |
| Valid | No. | 37 | 56.9 | 56.9 | 56.9 |
|  | Yes | 28 | 43.1 | 43.1 | 100.0 |
|  | Total | 65 | 100.0 | 100.0 |  |

| **Acinetobacter baumanii** | | | | | |
| --- | --- | --- | --- | --- | --- |
|  | | Frequency | Percent | Valid Percent | Cumulative Percent |
| Valid | No. | 25 | 38.5 | 89.3 | 89.3 |
|  | Yes | 3 | 4.6 | 10.7 | 100.0 |
|  | Total | 28 | 43.1 | 100.0 |  |
| Missing | System | 37 | 56.9 |  |  |
| Total | | 65 | 100.0 |  |  |

| **Escherichia coli** | | | | | |
| --- | --- | --- | --- | --- | --- |
|  | | Frequency | Percent | Valid Percent | Cumulative Percent |
| Valid | No. | 21 | 32.3 | 75.0 | 75.0 |
|  | Yes | 7 | 10.8 | 25.0 | 100.0 |
|  | Total | 28 | 43.1 | 100.0 |  |
| Missing | System | 37 | 56.9 |  |  |
| Total | | 65 | 100.0 |  |  |

| **Staphylococcus aureus** | | | | | |
| --- | --- | --- | --- | --- | --- |
|  | | Frequency | Percent | Valid Percent | Cumulative Percent |
| Valid | No. | 23 | 35.4 | 82.1 | 82.1 |
|  | Yes | 5 | 7.7 | 17.9 | 100.0 |
|  | Total | 28 | 43.1 | 100.0 |  |
| Missing | System | 37 | 56.9 |  |  |
| Total | | 65 | 100.0 |  |  |

| **Staphylococcus haemolyticus** | | | | | |
| --- | --- | --- | --- | --- | --- |
|  | | Frequency | Percent | Valid Percent | Cumulative Percent |
| Valid | No. | 26 | 40.0 | 92.9 | 92.9 |
|  | Yes | 2 | 3.1 | 7.1 | 100.0 |
|  | Total | 28 | 43.1 | 100.0 |  |
| Missing | System | 37 | 56.9 |  |  |
| Total | | 65 | 100.0 |  |  |

| **Staphylococcus epidermidis** | | | | | |
| --- | --- | --- | --- | --- | --- |
|  | | Frequency | Percent | Valid Percent | Cumulative Percent |
| Valid | No. | 27 | 41.5 | 96.4 | 96.4 |
|  | Yes | 1 | 1.5 | 3.6 | 100.0 |
|  | Total | 28 | 43.1 | 100.0 |  |
| Missing | System | 37 | 56.9 |  |  |
| Total | | 65 | 100.0 |  |  |

| **Staphylococcus sciuri** | | | | | |
| --- | --- | --- | --- | --- | --- |
|  | | Frequency | Percent | Valid Percent | Cumulative Percent |
| Valid | No. | 27 | 41.5 | 96.4 | 96.4 |
|  | Yes | 1 | 1.5 | 3.6 | 100.0 |
|  | Total | 28 | 43.1 | 100.0 |  |
| Missing | System | 37 | 56.9 |  |  |
| Total | | 65 | 100.0 |  |  |

| **Enterococcus faecalis** | | | | | |
| --- | --- | --- | --- | --- | --- |
|  | | Frequency | Percent | Valid Percent | Cumulative Percent |
| Valid | No. | 22 | 33.8 | 78.6 | 78.6 |
|  | Yes | 6 | 9.2 | 21.4 | 100.0 |
|  | Total | 28 | 43.1 | 100.0 |  |
| Missing | System | 37 | 56.9 |  |  |
| Total | | 65 | 100.0 |  |  |

| **Enterobacter cloacae** | | | | | |
| --- | --- | --- | --- | --- | --- |
|  | | Frequency | Percent | Valid Percent | Cumulative Percent |
| Valid | No. | 26 | 40.0 | 92.9 | 92.9 |
|  | Yes | 2 | 3.1 | 7.1 | 100.0 |
|  | Total | 28 | 43.1 | 100.0 |  |
| Missing | System | 37 | 56.9 |  |  |
| Total | | 65 | 100.0 |  |  |

| **Enterobacter aerogenes** | | | | | |
| --- | --- | --- | --- | --- | --- |
|  | | Frequency | Percent | Valid Percent | Cumulative Percent |
| Valid | No. | 27 | 41.5 | 96.4 | 96.4 |
|  | Yes | 1 | 1.5 | 3.6 | 100.0 |
|  | Total | 28 | 43.1 | 100.0 |  |
| Missing | System | 37 | 56.9 |  |  |
| Total | | 65 | 100.0 |  |  |

| **Streptococcus mitis** | | | | | |
| --- | --- | --- | --- | --- | --- |
|  | | Frequency | Percent | Valid Percent | Cumulative Percent |
| Valid | No. | 27 | 41.5 | 96.4 | 96.4 |
|  | Yes | 1 | 1.5 | 3.6 | 100.0 |
|  | Total | 28 | 43.1 | 100.0 |  |
| Missing | System | 37 | 56.9 |  |  |
| Total | | 65 | 100.0 |  |  |

| **Klebsiella aerogenes** | | | | | |
| --- | --- | --- | --- | --- | --- |
|  | | Frequency | Percent | Valid Percent | Cumulative Percent |
| Valid | No. | 26 | 40.0 | 92.9 | 92.9 |
|  | Yes | 2 | 3.1 | 7.1 | 100.0 |
|  | Total | 28 | 43.1 | 100.0 |  |
| Missing | System | 37 | 56.9 |  |  |
| Total | | 65 | 100.0 |  |  |

| **Pantoea agglomerans** | | | | | |
| --- | --- | --- | --- | --- | --- |
|  | | Frequency | Percent | Valid Percent | Cumulative Percent |
| Valid | No. | 27 | 41.5 | 96.4 | 96.4 |
|  | Yes | 1 | 1.5 | 3.6 | 100.0 |
|  | Total | 28 | 43.1 | 100.0 |  |
| Missing | System | 37 | 56.9 |  |  |
| Total | | 65 | 100.0 |  |  |

| **Pseudomonas putida** | | | | | |
| --- | --- | --- | --- | --- | --- |
|  | | Frequency | Percent | Valid Percent | Cumulative Percent |
| Valid | No. | 27 | 41.5 | 96.4 | 96.4 |
|  | Yes | 1 | 1.5 | 3.6 | 100.0 |
|  | Total | 28 | 43.1 | 100.0 |  |
| Missing | System | 37 | 56.9 |  |  |
| Total | | 65 | 100.0 |  |  |

| **proteus vulgaris** | | | | | |
| --- | --- | --- | --- | --- | --- |
|  | | Frequency | Percent | Valid Percent | Cumulative Percent |
| Valid | No. | 27 | 41.5 | 96.4 | 96.4 |
|  | Yes | 1 | 1.5 | 3.6 | 100.0 |
|  | Total | 28 | 43.1 | 100.0 |  |
| Missing | System | 37 | 56.9 |  |  |
| Total | | 65 | 100.0 |  |  |

| **candida tropicalis** | | | | | |
| --- | --- | --- | --- | --- | --- |
|  | | Frequency | Percent | Valid Percent | Cumulative Percent |
| Valid | No. | 27 | 41.5 | 96.4 | 96.4 |
|  | Yes | 1 | 1.5 | 3.6 | 100.0 |
|  | Total | 28 | 43.1 | 100.0 |  |
| Missing | System | 37 | 56.9 |  |  |
| Total | | 65 | 100.0 |  |  |

| **Positivity** | | | | | |
| --- | --- | --- | --- | --- | --- |
|  | | Frequency | Percent | Valid Percent | Cumulative Percent |
| Valid | Clinically -, Culture - | 33 | 50.8 | 50.8 | 50.8 |
|  | Clinically +, Culture + | 28 | 43.1 | 43.1 | 93.8 |
|  | Clinically +, Culture - | 4 | 6.2 | 6.2 | 100.0 |
|  | Total | 65 | 100.0 | 100.0 |  |

| **SSI degree** | | | | | |
| --- | --- | --- | --- | --- | --- |
|  | | Frequency | Percent | Valid Percent | Cumulative Percent |
| Valid | No. | 33 | 50.8 | 50.8 | 50.8 |
|  | Deep | 6 | 9.2 | 9.2 | 60.0 |
|  | Superficial | 26 | 40.0 | 40.0 | 100.0 |
|  | Total | 65 | 100.0 | 100.0 |  |

**Frequencies**

| **Statistics** | | |
| --- | --- | --- |
| Usia (tahun) | | |
| N | Valid | 65 |
|  | Missing | 0 |
| Mean | | 6.4438 |
| Median | | 5.2500 |
| Std. Deviation | | 4.56792 |
| Minimum | | .00 |
| Maximum | | 18.00 |
| Percentiles | 25 | 2.8500 |
|  | 50 | 5.2500 |
|  | 75 | 10.0000 |

**Explore**

| **Tests of Normality** | | | | | | |
| --- | --- | --- | --- | --- | --- | --- |
|  | Kolmogorov-Smirnov^a^ | | | Shapiro-Wilk | | |
|  | Statistic | df | Sig. | Statistic | df | Sig. |
| Usia (tahun) | .123 | 65 | .016 | .927 | 65 | .001 |
| a. Lilliefors Significance Correction | | | | | | |

**Crosstabs**

**Urethral meatys location * edema**

| **Crosstab** | | | | | |
| --- | --- | --- | --- | --- | --- |
|  | | | edema | | Total |
|  |  |  | Tidak | Ya |  |
| Urethral meatus location | Proximal | Count | 24 | 14 | 38 |
|  |  | % within urethral meatus location | 63.2% | 36.8% | 100.0% |
|  |  | % within edema | 55.8% | 63.6% | 58.5% |
|  | Distal | Count | 19 | 8 | 27 |
|  |  | % within urethral meatus location | 70.4% | 29.6% | 100.0% |
|  |  | % within edema | 44.2% | 36.4% | 41.5% |
| Total | | Count | 43 | 22 | 65 |
|  |  | % within urethral meatus location | 66.2% | 33.8% | 100.0% |
|  |  | % within edema | 100.0% | 100.0% | 100.0% |

| **Chi-Square Tests** | | | | | |
| --- | --- | --- | --- | --- | --- |
|  | Value | df | Asymptotic Significance (2-sided) | Exact Sig. (2-sided) | Exact Sig. (1-sided) |
| Pearson Chi-Square | .367^a^ | 1 | .545 |  |  |
| Continuity Correction^b^ | .115 | 1 | .734 |  |  |
| Likelihood Ratio | .369 | 1 | .543 |  |  |
| Fisher's Exact Test |  |  |  | .603 | .369 |
| Linear-by-Linear Association | .361 | 1 | .548 |  |  |
| N of Valid Cases | 65 |  |  |  |  |
| a. 0 cells (.0%) have expected count less than 5. The minimum expected count is 9.14. | | | | | |
| b. Computed only for a 2x2 table | | | | | |

**Urethral meatus location * Bleeding**

| **Crosstab** | | | | | |
| --- | --- | --- | --- | --- | --- |
|  | | | Bleeding | | Total |
|  |  |  | No | Yes |  |
| Urethral meatus location | Proximal | Count | 34 | 4 | 38 |
|  |  | % within urethral meatus location | 89.5% | 10.5% | 100.0% |
|  |  | % within bleeding | 58.6% | 57.1% | 58.5% |
|  | Distal | Count | 24 | 3 | 27 |
|  |  | % within urethral meatus location | 88.9% | 11.1% | 100.0% |
|  |  | % within bleeding | 41.4% | 42.9% | 41.5% |
| Total | | Count | 58 | 7 | 65 |
|  |  | % within urethral meatus location | 89.2% | 10.8% | 100.0% |
|  |  | % within Bleeding | 100.0% | 100.0% | 100.0% |

| **Chi-Square Tests** | | | | | |
| --- | --- | --- | --- | --- | --- |
|  | Value | df | Asymptotic Significance (2-sided) | Exact Sig. (2-sided) | Exact Sig. (1-sided) |
| Pearson Chi-Square | .006^a^ | 1 | .940 |  |  |
| Continuity Correction^b^ | .000 | 1 | 1.000 |  |  |
| Likelihood Ratio | .006 | 1 | .940 |  |  |
| Fisher's Exact Test |  |  |  | 1.000 | .622 |
| Linear-by-Linear Association | .006 | 1 | .941 |  |  |
| N of Valid Cases | 65 |  |  |  |  |
| a. 2 cells (50.0%) have expected count less than 5. The minimum expected count is 2.91. | | | | | |
| b. Computed only for a 2x2 table | | | | | |

**Urethral meatus location * Pus**

| **Crosstab** | | | | | |
| --- | --- | --- | --- | --- | --- |
|  | | | Pus | | Total |
|  |  |  | Tidak | Ya |  |
| Urethral meatus location | Proximal | Count | 33 | 5 | 38 |
|  |  | % within urethral meatus location | 86.8% | 13.2% | 100.0% |
|  |  | % within Pus | 56.9% | 71.4% | 58.5% |
|  | Distal | Count | 25 | 2 | 27 |
|  |  | % within urethral meatus location | 92.6% | 7.4% | 100.0% |
|  |  | % within Pus | 43.1% | 28.6% | 41.5% |
| Total | | Count | 58 | 7 | 65 |
|  |  | % within urethral meatus location | 89.2% | 10.8% | 100.0% |
|  |  | % within Pus | 100.0% | 100.0% | 100.0% |

| **Chi-Square Tests** | | | | | |
| --- | --- | --- | --- | --- | --- |
|  | Value | df | Asymptotic Significance (2-sided) | Exact Sig. (2-sided) | Exact Sig. (1-sided) |
| Pearson Chi-Square | .543^a^ | 1 | .461 |  |  |
| Continuity Correction^b^ | .110 | 1 | .741 |  |  |
| Likelihood Ratio | .565 | 1 | .452 |  |  |
| Fisher's Exact Test |  |  |  | .690 | .378 |
| Linear-by-Linear Association | .535 | 1 | .465 |  |  |
| N of Valid Cases | 65 |  |  |  |  |
| a. 2 cells (50.0%) have expected count less than 5. The minimum expected count is 2.91. | | | | | |
| b. Computed only for a 2x2 table | | | | | |

**Urethral meatus location * Dehiscence**

| **Crosstab** | | | | | |
| --- | --- | --- | --- | --- | --- |
|  | | | Dehiscence | | Total |
|  |  |  | No | Yes |  |
| Urethral meatus location | Proximal | Count | 34 | 4 | 38 |
|  |  | % within urethral meatus location | 89.5% | 10.5% | 100.0% |
|  |  | % within Dehiscence | 57.6% | 66.7% | 58.5% |
|  | Distal | Count | 25 | 2 | 27 |
|  |  | % within urethral meatus location | 92.6% | 7.4% | 100.0% |
|  |  | % within Dehiscence | 42.4% | 33.3% | 41.5% |
| Total | | Count | 59 | 6 | 65 |
|  |  | % within urethral meatus location | 90.8% | 9.2% | 100.0% |
|  |  | % within Dehiscence | 100.0% | 100.0% | 100.0% |

| **Chi-Square Tests** | | | | | |
| --- | --- | --- | --- | --- | --- |
|  | Value | df | Asymptotic Significance (2-sided) | Exact Sig. (2-sided) | Exact Sig. (1-sided) |
| Pearson Chi-Square | .183^a^ | 1 | .669 |  |  |
| Continuity Correction^b^ | .000 | 1 | 1.000 |  |  |
| Likelihood Ratio | .187 | 1 | .665 |  |  |
| Fisher's Exact Test |  |  |  | 1.000 | .511 |
| Linear-by-Linear Association | .180 | 1 | .671 |  |  |
| N of Valid Cases | 65 |  |  |  |  |
| a. 2 cells (50.0%) have expected count less than 5. The minimum expected count is 2.49. | | | | | |
| b. Computed only for a 2x2 table | | | | | |

**Urethral meatus location * Fistula**

| **Crosstab** | | | | | |
| --- | --- | --- | --- | --- | --- |
|  | | | Fistula | | Total |
|  |  |  | Tidak | Ya |  |
| Urethral meatus location | Proximal | Count | 35 | 3 | 38 |
|  |  | % within Urethral meatus location | 92.1% | 7.9% | 100.0% |
|  |  | % within Fistula | 58.3% | 60.0% | 58.5% |
|  | Distal | Count | 25 | 2 | 27 |
|  |  | % within Urethral meatus location | 92.6% | 7.4% | 100.0% |
|  |  | % within Fistula | 41.7% | 40.0% | 41.5% |
| Total | | Count | 60 | 5 | 65 |
|  |  | % within Urethral meatus location | 92.3% | 7.7% | 100.0% |
|  |  | % within Fistula | 100.0% | 100.0% | 100.0% |

| **Chi-Square Tests** | | | | | |
| --- | --- | --- | --- | --- | --- |
|  | Value | df | Asymptotic Significance (2-sided) | Exact Sig. (2-sided) | Exact Sig. (1-sided) |
| Pearson Chi-Square | .005^a^ | 1 | .942 |  |  |
| Continuity Correction^b^ | .000 | 1 | 1.000 |  |  |
| Likelihood Ratio | .005 | 1 | .942 |  |  |
| Fisher's Exact Test |  |  |  | 1.000 | .661 |
| Linear-by-Linear Association | .005 | 1 | .943 |  |  |
| N of Valid Cases | 65 |  |  |  |  |
| a. 2 cells (50.0%) have expected count less than 5. The minimum expected count is 2.08. | | | | | |
| b. Computed only for a 2x2 table | | | | | |

**Urethral meatus location * SSI clinically +**

| **Crosstab** | | | | | |
| --- | --- | --- | --- | --- | --- |
|  | | | Klinis IDO + | | Total |
|  |  |  | Tidak | Ya |  |
| Urethral meatus location | Proximal | Count | 15 | 23 | 38 |
|  |  | % within Urethral meatus location | 39.5% | 60.5% | 100.0% |
|  |  | % within clinically SSI + | 45.5% | 71.9% | 58.5% |
|  | Distal | Count | 18 | 9 | 27 |
|  |  | % within Urethral meatus location | 66.7% | 33.3% | 100.0% |
|  |  | % within clinically SSI + | 54.5% | 28.1% | 41.5% |
| Total | | Count | 33 | 32 | 65 |
|  |  | % within Urethral meatus location | 50.8% | 49.2% | 100.0% |
|  |  | % within clinically SSI + | 100.0% | 100.0% | 100.0% |

| **Chi-Square Tests** | | | | | |
| --- | --- | --- | --- | --- | --- |
|  | Value | df | Asymptotic Significance (2-sided) | Exact Sig. (2-sided) | Exact Sig. (1-sided) |
| Pearson Chi-Square | 4.670^a^ | 1 | .031 |  |  |
| Continuity Correction^b^ | 3.645 | 1 | .056 |  |  |
| Likelihood Ratio | 4.740 | 1 | .029 |  |  |
| Fisher's Exact Test |  |  |  | .044 | .028 |
| Linear-by-Linear Association | 4.598 | 1 | .032 |  |  |
| N of Valid Cases | 65 |  |  |  |  |
| a. 0 cells (.0%) have expected count less than 5. The minimum expected count is 13.29. | | | | | |
| b. Computed only for a 2x2 table | | | | | |

**Urethral meatus location * positive culture**

| **Crosstab** | | | | | |
| --- | --- | --- | --- | --- | --- |
|  | | | Kultur Positif | | Total |
|  |  |  | Tidak | Ya |  |
| Lokasi meatus uretra | Proksimal | Count | 17 | 21 | 38 |
|  |  | % within Urethral meatus location | 44.7% | 55.3% | 100.0% |
|  |  | % within positive culture | 45.9% | 75.0% | 58.5% |
|  | Distal | Count | 20 | 7 | 27 |
|  |  | % within Urethral meatus location | 74.1% | 25.9% | 100.0% |
|  |  | % within positive culture | 54.1% | 25.0% | 41.5% |
| Total | | Count | 37 | 28 | 65 |
|  |  | % within Urethral meatus location | 56.9% | 43.1% | 100.0% |
|  |  | % within positive culture | 100.0% | 100.0% | 100.0% |

| **Chi-Square Tests** | | | | | |
| --- | --- | --- | --- | --- | --- |
|  | Value | df | Asymptotic Significance (2-sided) | Exact Sig. (2-sided) | Exact Sig. (1-sided) |
| Pearson Chi-Square | 5.540^a^ | 1 | .019 |  |  |
| Continuity Correction^b^ | 4.409 | 1 | .036 |  |  |
| Likelihood Ratio | 5.698 | 1 | .017 |  |  |
| Fisher's Exact Test |  |  |  | .024 | .017 |
| Linear-by-Linear Association | 5.455 | 1 | .020 |  |  |
| N of Valid Cases | 65 |  |  |  |  |
| a. 0 cells (.0%) have expected count less than 5. The minimum expected count is 11.63. | | | | | |
| b. Computed only for a 2x2 table | | | | | |

**Urethral meatus location * Positivity**

| **Crosstab** | | | | | | |
| --- | --- | --- | --- | --- | --- | --- |
|  | | | Positivitas | | | Total |
|  |  |  | Klinis -, Kultur - | Klinis +, Kultur + | Klinis +, Kultur - |  |
| Urethral meatus location | Proksimal | Count | 15 | 21 | 2 | 38 |
|  |  | % within Urethral meatus location | 39.5% | 55.3% | 5.3% | 100.0% |
|  |  | % within Positivity | 45.5% | 75.0% | 50.0% | 58.5% |
|  | Distal | Count | 18 | 7 | 2 | 27 |
|  |  | % within Urethral meatus location | 66.7% | 25.9% | 7.4% | 100.0% |
|  |  | % within Positivity | 54.5% | 25.0% | 50.0% | 41.5% |
| Total | | Count | 33 | 28 | 4 | 65 |
|  |  | % within Urethral meatus location | 50.8% | 43.1% | 6.2% | 100.0% |
|  |  | % within Positivity | 100.0% | 100.0% | 100.0% | 100.0% |

| **Chi-Square Tests** | | | |
| --- | --- | --- | --- |
|  | Value | df | Asymptotic Significance (2-sided) |
| Pearson Chi-Square | 5.571^a^ | 2 | .062 |
| Likelihood Ratio | 5.728 | 2 | .057 |
| Linear-by-Linear Association | 2.634 | 1 | .105 |
| N of Valid Cases | 65 |  |  |
| a. 2 cells (33.3%) have expected count less than 5. The minimum expected count is 1.66. | | | |

**Lokasi meatus uretra * Derajat IDO**

| **Crosstab** | | | | | | |
| --- | --- | --- | --- | --- | --- | --- |
|  | | | Derajat IDO | | | Total |
|  |  |  | Tidak | Dalam | Superfisial |  |
| Lokasi meatus uretra | Proksimal | Count | 15 | 4 | 19 | 38 |
|  |  | % within Lokasi meatus uretra | 39.5% | 10.5% | 50.0% | 100.0% |
|  |  | % within Derajat IDO | 45.5% | 66.7% | 73.1% | 58.5% |
|  | Distal | Count | 18 | 2 | 7 | 27 |
|  |  | % within Lokasi meatus uretra | 66.7% | 7.4% | 25.9% | 100.0% |
|  |  | % within Derajat IDO | 54.5% | 33.3% | 26.9% | 41.5% |
| Total | | Count | 33 | 6 | 26 | 65 |
|  |  | % within Lokasi meatus uretra | 50.8% | 9.2% | 40.0% | 100.0% |
|  |  | % within Derajat IDO | 100.0% | 100.0% | 100.0% | 100.0% |

| **Chi-Square Tests** | | | |
| --- | --- | --- | --- |
|  | Value | df | Asymptotic Significance (2-sided) |
| Pearson Chi-Square | 4.752^a^ | 2 | .093 |
| Likelihood Ratio | 4.836 | 2 | .089 |
| Linear-by-Linear Association | 4.559 | 1 | .033 |
| N of Valid Cases | 65 |  |  |
| a. 2 cells (33.3%) have expected count less than 5. The minimum expected count is 2.49. | | | |

**Tindakan * edema**

| **Crosstab** | | | | | |
| --- | --- | --- | --- | --- | --- |
|  | | | edema | | Total |
|  |  |  | Tidak | Ya |  |
| Tindakan | Uretroplasti | Count | 33 | 17 | 50 |
|  |  | % within Tindakan | 66.0% | 34.0% | 100.0% |
|  |  | % within edema | 76.7% | 77.3% | 76.9% |
|  | Kordektomi | Count | 10 | 5 | 15 |
|  |  | % within Tindakan | 66.7% | 33.3% | 100.0% |
|  |  | % within edema | 23.3% | 22.7% | 23.1% |
| Total | | Count | 43 | 22 | 65 |
|  |  | % within Tindakan | 66.2% | 33.8% | 100.0% |
|  |  | % within edema | 100.0% | 100.0% | 100.0% |

| **Chi-Square Tests** | | | | | |
| --- | --- | --- | --- | --- | --- |
|  | Value | df | Asymptotic Significance (2-sided) | Exact Sig. (2-sided) | Exact Sig. (1-sided) |
| Pearson Chi-Square | .002^a^ | 1 | .962 |  |  |
| Continuity Correction^b^ | .000 | 1 | 1.000 |  |  |
| Likelihood Ratio | .002 | 1 | .962 |  |  |
| Fisher's Exact Test |  |  |  | 1.000 | .610 |
| Linear-by-Linear Association | .002 | 1 | .962 |  |  |
| N of Valid Cases | 65 |  |  |  |  |
| a. 0 cells (.0%) have expected count less than 5. The minimum expected count is 5.08. | | | | | |
| b. Computed only for a 2x2 table | | | | | |

**Tindakan * Perdarahan**

| **Crosstab** | | | | | |
| --- | --- | --- | --- | --- | --- |
|  | | | Perdarahan | | Total |
|  |  |  | Tidak | Ya |  |
| Tindakan | Uretroplasti | Count | 45 | 5 | 50 |
|  |  | % within Tindakan | 90.0% | 10.0% | 100.0% |
|  |  | % within Perdarahan | 77.6% | 71.4% | 76.9% |
|  | Kordektomi | Count | 13 | 2 | 15 |
|  |  | % within Tindakan | 86.7% | 13.3% | 100.0% |
|  |  | % within Perdarahan | 22.4% | 28.6% | 23.1% |
| Total | | Count | 58 | 7 | 65 |
|  |  | % within Tindakan | 89.2% | 10.8% | 100.0% |
|  |  | % within Perdarahan | 100.0% | 100.0% | 100.0% |

| **Chi-Square Tests** | | | | | |
| --- | --- | --- | --- | --- | --- |
|  | Value | df | Asymptotic Significance (2-sided) | Exact Sig. (2-sided) | Exact Sig. (1-sided) |
| Pearson Chi-Square | .133^a^ | 1 | .715 |  |  |
| Continuity Correction^b^ | .000 | 1 | 1.000 |  |  |
| Likelihood Ratio | .128 | 1 | .721 |  |  |
| Fisher's Exact Test |  |  |  | .658 | .514 |
| Linear-by-Linear Association | .131 | 1 | .717 |  |  |
| N of Valid Cases | 65 |  |  |  |  |
| a. 1 cells (25.0%) have expected count less than 5. The minimum expected count is 1.62. | | | | | |
| b. Computed only for a 2x2 table | | | | | |

**Tindakan * Pus**

| **Crosstab** | | | | | |
| --- | --- | --- | --- | --- | --- |
|  | | | Pus | | Total |
|  |  |  | Tidak | Ya |  |
| Tindakan | Uretroplasti | Count | 44 | 6 | 50 |
|  |  | % within Tindakan | 88.0% | 12.0% | 100.0% |
|  |  | % within Pus | 75.9% | 85.7% | 76.9% |
|  | Kordektomi | Count | 14 | 1 | 15 |
|  |  | % within Tindakan | 93.3% | 6.7% | 100.0% |
|  |  | % within Pus | 24.1% | 14.3% | 23.1% |
| Total | | Count | 58 | 7 | 65 |
|  |  | % within Tindakan | 89.2% | 10.8% | 100.0% |
|  |  | % within Pus | 100.0% | 100.0% | 100.0% |

| **Chi-Square Tests** | | | | | |
| --- | --- | --- | --- | --- | --- |
|  | Value | df | Asymptotic Significance (2-sided) | Exact Sig. (2-sided) | Exact Sig. (1-sided) |
| Pearson Chi-Square | .342^a^ | 1 | .559 |  |  |
| Continuity Correction^b^ | .012 | 1 | .913 |  |  |
| Likelihood Ratio | .376 | 1 | .540 |  |  |
| Fisher's Exact Test |  |  |  | 1.000 | .486 |
| Linear-by-Linear Association | .336 | 1 | .562 |  |  |
| N of Valid Cases | 65 |  |  |  |  |
| a. 1 cells (25.0%) have expected count less than 5. The minimum expected count is 1.62. | | | | | |
| b. Computed only for a 2x2 table | | | | | |

**Tindakan * Dehisens**

| **Crosstab** | | | | | |
| --- | --- | --- | --- | --- | --- |
|  | | | Dehisens | | Total |
|  |  |  | Tidak | Ya |  |
| Tindakan | Uretroplasti | Count | 45 | 5 | 50 |
|  |  | % within Tindakan | 90.0% | 10.0% | 100.0% |
|  |  | % within Dehisens | 76.3% | 83.3% | 76.9% |
|  | Kordektomi | Count | 14 | 1 | 15 |
|  |  | % within Tindakan | 93.3% | 6.7% | 100.0% |
|  |  | % within Dehisens | 23.7% | 16.7% | 23.1% |
| Total | | Count | 59 | 6 | 65 |
|  |  | % within Tindakan | 90.8% | 9.2% | 100.0% |
|  |  | % within Dehisens | 100.0% | 100.0% | 100.0% |

| **hi-Square Tests** | | | | | |
| --- | --- | --- | --- | --- | --- |
|  | Value | df | Asymptotic Significance (2-sided) | Exact Sig. (2-sided) | Exact Sig. (1-sided) |
| Pearson Chi-Square | .153^a^ | 1 | .696 |  |  |
| Continuity Correction^b^ | .000 | 1 | 1.000 |  |  |
| Likelihood Ratio | .164 | 1 | .686 |  |  |
| Fisher's Exact Test |  |  |  | 1.000 | .577 |
| Linear-by-Linear Association | .151 | 1 | .698 |  |  |
| N of Valid Cases | 65 |  |  |  |  |
| a. 2 cells (50.0%) have expected count less than 5. The minimum expected count is 1.38. | | | | | |
| b. Computed only for a 2x2 table | | | | | |

**Tindakan * Fistula**

| **Crosstab** | | | | | |
| --- | --- | --- | --- | --- | --- |
|  | | | Fistula | | Total |
|  |  |  | Tidak | Ya |  |
| Tindakan | Uretroplasti | Count | 45 | 5 | 50 |
|  |  | % within Tindakan | 90.0% | 10.0% | 100.0% |
|  |  | % within Fistula | 75.0% | 100.0% | 76.9% |
|  | Kordektomi | Count | 15 | 0 | 15 |
|  |  | % within Tindakan | 100.0% | 0.0% | 100.0% |
|  |  | % within Fistula | 25.0% | 0.0% | 23.1% |
| Total | | Count | 60 | 5 | 65 |
|  |  | % within Tindakan | 92.3% | 7.7% | 100.0% |
|  |  | % within Fistula | 100.0% | 100.0% | 100.0% |

| **Chi-Square Tests** | | | | | |
| --- | --- | --- | --- | --- | --- |
|  | Value | df | Asymptotic Significance (2-sided) | Exact Sig. (2-sided) | Exact Sig. (1-sided) |
| Pearson Chi-Square | 1.625^a^ | 1 | .202 |  |  |
| Continuity Correction^b^ | .522 | 1 | .470 |  |  |
| Likelihood Ratio | 2.746 | 1 | .097 |  |  |
| Fisher's Exact Test |  |  |  | .582 | .257 |
| Linear-by-Linear Association | 1.600 | 1 | .206 |  |  |
| N of Valid Cases | 65 |  |  |  |  |
| a. 2 cells (50.0%) have expected count less than 5. The minimum expected count is 1.15. | | | | | |
| b. Computed only for a 2x2 table | | | | | |

**Tindakan * Klinis IDO +**

| **Crosstab** | | | | | |
| --- | --- | --- | --- | --- | --- |
|  | | | Klinis IDO + | | Total |
|  |  |  | Tidak | Ya |  |
| Tindakan | Uretroplasti | Count | 27 | 23 | 50 |
|  |  | % within Tindakan | 54.0% | 46.0% | 100.0% |
|  |  | % within Klinis IDO + | 81.8% | 71.9% | 76.9% |
|  | Kordektomi | Count | 6 | 9 | 15 |
|  |  | % within Tindakan | 40.0% | 60.0% | 100.0% |
|  |  | % within Klinis IDO + | 18.2% | 28.1% | 23.1% |
| Total | | Count | 33 | 32 | 65 |
|  |  | % within Tindakan | 50.8% | 49.2% | 100.0% |
|  |  | % within Klinis IDO + | 100.0% | 100.0% | 100.0% |

| **Chi-Square Tests** | | | | | |
| --- | --- | --- | --- | --- | --- |
|  | Value | df | Asymptotic Significance (2-sided) | Exact Sig. (2-sided) | Exact Sig. (1-sided) |
| Pearson Chi-Square | .905^a^ | 1 | .341 |  |  |
| Continuity Correction^b^ | .431 | 1 | .511 |  |  |
| Likelihood Ratio | .909 | 1 | .340 |  |  |
| Fisher's Exact Test |  |  |  | .389 | .256 |
| Linear-by-Linear Association | .891 | 1 | .345 |  |  |
| N of Valid Cases | 65 |  |  |  |  |
| a. 0 cells (.0%) have expected count less than 5. The minimum expected count is 7.38. | | | | | |
| b. Computed only for a 2x2 table | | | | | |

**Tindakan * Kultur Positif**

| **Crosstab** | | | | | |
| --- | --- | --- | --- | --- | --- |
|  | | | Kultur Positif | | Total |
|  |  |  | Tidak | Ya |  |
| Tindakan | Uretroplasti | Count | 31 | 19 | 50 |
|  |  | % within Tindakan | 62.0% | 38.0% | 100.0% |
|  |  | % within Kultur Positif | 83.8% | 67.9% | 76.9% |
|  | Kordektomi | Count | 6 | 9 | 15 |
|  |  | % within Tindakan | 40.0% | 60.0% | 100.0% |
|  |  | % within Kultur Positif | 16.2% | 32.1% | 23.1% |
| Total | | Count | 37 | 28 | 65 |
|  |  | % within Tindakan | 56.9% | 43.1% | 100.0% |
|  |  | % within Kultur Positif | 100.0% | 100.0% | 100.0% |

| **Chi-Square Tests** | | | | | |
| --- | --- | --- | --- | --- | --- |
|  | Value | df | Asymptotic Significance (2-sided) | Exact Sig. (2-sided) | Exact Sig. (1-sided) |
| Pearson Chi-Square | 2.278^a^ | 1 | .131 |  |  |
| Continuity Correction^b^ | 1.469 | 1 | .226 |  |  |
| Likelihood Ratio | 2.262 | 1 | .133 |  |  |
| Fisher's Exact Test |  |  |  | .150 | .113 |
| Linear-by-Linear Association | 2.242 | 1 | .134 |  |  |
| N of Valid Cases | 65 |  |  |  |  |
| a. 0 cells (.0%) have expected count less than 5. The minimum expected count is 6.46. | | | | | |
| b. Computed only for a 2x2 table | | | | | |

**Tindakan * Positivitas**

| **Crosstab** | | | | | | |
| --- | --- | --- | --- | --- | --- | --- |
|  | | | Positivitas | | | Total |
|  |  |  | Klinis -, Kultur - | Klinis +, Kultur + | Klinis +, Kultur - |  |
| Tindakan | Uretroplasti | Count | 27 | 19 | 4 | 50 |
|  |  | % within Tindakan | 54.0% | 38.0% | 8.0% | 100.0% |
|  |  | % within Positivitas | 81.8% | 67.9% | 100.0% | 76.9% |
|  | Kordektomi | Count | 6 | 9 | 0 | 15 |
|  |  | % within Tindakan | 40.0% | 60.0% | 0.0% | 100.0% |
|  |  | % within Positivitas | 18.2% | 32.1% | 0.0% | 23.1% |
| Total | | Count | 33 | 28 | 4 | 65 |
|  |  | % within Tindakan | 50.8% | 43.1% | 6.2% | 100.0% |
|  |  | % within Positivitas | 100.0% | 100.0% | 100.0% | 100.0% |

| **Chi-Square Tests** | | | |
| --- | --- | --- | --- |
|  | Value | df | Asymptotic Significance (2-sided) |
| Pearson Chi-Square | 2.942^a^ | 2 | .230 |
| Likelihood Ratio | 3.769 | 2 | .152 |
| Linear-by-Linear Association | .110 | 1 | .740 |
| N of Valid Cases | 65 |  |  |
| a. 2 cells (33.3%) have expected count less than 5. The minimum expected count is .92. | | | |

**Tindakan * Derajat IDO**

| **Crosstab** | | | | | | |
| --- | --- | --- | --- | --- | --- | --- |
|  | | | Derajat IDO | | | Total |
|  |  |  | Tidak | Dalam | Superfisial |  |
| Tindakan | Uretroplasti | Count | 27 | 5 | 18 | 50 |
|  |  | % within Tindakan | 54.0% | 10.0% | 36.0% | 100.0% |
|  |  | % within Derajat IDO | 81.8% | 83.3% | 69.2% | 76.9% |
|  | Kordektomi | Count | 6 | 1 | 8 | 15 |
|  |  | % within Tindakan | 40.0% | 6.7% | 53.3% | 100.0% |
|  |  | % within Derajat IDO | 18.2% | 16.7% | 30.8% | 23.1% |
| Total | | Count | 33 | 6 | 26 | 65 |
|  |  | % within Tindakan | 50.8% | 9.2% | 40.0% | 100.0% |
|  |  | % within Derajat IDO | 100.0% | 100.0% | 100.0% | 100.0% |

| **Chi-Square Tests** | | | |
| --- | --- | --- | --- |
|  | Value | df | Asymptotic Significance (2-sided) |
| Pearson Chi-Square | 1.451^a^ | 2 | .484 |
| Likelihood Ratio | 1.430 | 2 | .489 |
| Linear-by-Linear Association | 1.245 | 1 | .265 |
| N of Valid Cases | 65 |  |  |
| a. 2 cells (33.3%) have expected count less than 5. The minimum expected count is 1.38. | | | |

**Explore**

| **Descriptives** | | | | | |
| --- | --- | --- | --- | --- | --- |
|  | Klinis IDO + | | | Statistic | Std. Error |
| Usia (tahun) | Tidak | Mean | | 6.2773 | .71660 |
|  |  | 95% Confidence Interval for Mean | Lower Bound | 4.8176 |  |
|  |  |  | Upper Bound | 7.7369 |  |
|  |  | 5% Trimmed Mean | | 6.0120 |  |
|  |  | Median | | 5.0000 |  |
|  |  | Variance | | 16.946 |  |
|  |  | Std. Deviation | | 4.11657 |  |
|  |  | Minimum | | .00 |  |
|  |  | Maximum | | 18.00 |  |
|  |  | Range | | 18.00 |  |
|  |  | Interquartile Range | | 3.95 |  |
|  |  | Skewness | | 1.238 | .409 |
|  |  | Kurtosis | | 1.591 | .798 |
|  | Ya | Mean | | 6.6156 | .89312 |
|  |  | 95% Confidence Interval for Mean | Lower Bound | 4.7941 |  |
|  |  |  | Upper Bound | 8.4372 |  |
|  |  | 5% Trimmed Mean | | 6.3472 |  |
|  |  | Median | | 5.5500 |  |
|  |  | Variance | | 25.525 |  |
|  |  | Std. Deviation | | 5.05225 |  |
|  |  | Minimum | | .50 |  |
|  |  | Maximum | | 17.60 |  |
|  |  | Range | | 17.10 |  |
|  |  | Interquartile Range | | 8.00 |  |
|  |  | Skewness | | .592 | .414 |
|  |  | Kurtosis | | -.730 | .809 |

| **Percentiles** | | | | | | | | | |
| --- | --- | --- | --- | --- | --- | --- | --- | --- | --- |
|  |  | Klinis IDO + | Percentiles | | | | | | |
|  |  |  | 5 | 10 | 25 | 50 | 75 | 90 | 95 |
| Weighted Average(Definition 1) | Usia (tahun) | Tidak | .2100 | 2.0000 | 4.0000 | 5.0000 | 7.9500 | 13.4400 | 16.6000 |
|  |  | Ya | .8250 | 1.0300 | 2.0000 | 5.5500 | 10.0000 | 13.8500 | 17.2100 |
| Tukey's Hinges | Usia (tahun) | Tidak |  |  | 4.0000 | 5.0000 | 7.9000 |  |  |
|  |  | Ya |  |  | 2.0000 | 5.5500 | 10.0000 |  |  |

| **Tests of Normality** | | | | | | | |
| --- | --- | --- | --- | --- | --- | --- | --- |
|  | Klinis IDO + | Kolmogorov-Smirnov^a^ | | | Shapiro-Wilk | | |
|  |  | Statistic | df | Sig. | Statistic | df | Sig. |
| Usia (tahun) | Tidak | .163 | 33 | .026 | .891 | 33 | .003 |
|  | Ya | .169 | 32 | .021 | .912 | 32 | .013 |
| a. Lilliefors Significance Correction | | | | | | | |

**Usia (tahun)**

**NPar Tests**

**Mann-Whitney Test**

| **Ranks** | | | | |
| --- | --- | --- | --- | --- |
|  | Klinis IDO + | N | Mean Rank | Sum of Ranks |
| Usia (tahun) | Tidak | 33 | 33.08 | 1091.50 |
|  | Ya | 32 | 32.92 | 1053.50 |
|  | Total | 65 |  |  |

| **Test Statistics^a^** | |
| --- | --- |
|  | Usia (tahun) |
| Mann-Whitney U | 525.500 |
| Wilcoxon W | 1053.500 |
| Z | -.033 |
| Asymp. Sig. (2-tailed) | .974 |
| a. Grouping Variable: Klinis IDO + | |

**Explore**

**edema**

| **Case Processing Summary** | | | | | | | |
| --- | --- | --- | --- | --- | --- | --- | --- |
|  | edema | Cases | | | | | |
|  |  | Valid | | Missing | | Total | |
|  |  | N | Percent | N | Percent | N | Percent |
| Usia (tahun) | Tidak | 43 | 100.0% | 0 | 0.0% | 43 | 100.0% |
|  | Ya | 22 | 100.0% | 0 | 0.0% | 22 | 100.0% |

| **Descriptives** | | | | | |
| --- | --- | --- | --- | --- | --- |
|  | edema | | | Statistic | Std. Error |
| Usia (tahun) | Tidak | Mean | | 6.5547 | .71107 |
|  |  | 95% Confidence Interval for Mean | Lower Bound | 5.1197 |  |
|  |  |  | Upper Bound | 7.9897 |  |
|  |  | 5% Trimmed Mean | | 6.2842 |  |
|  |  | Median | | 5.5000 |  |
|  |  | Variance | | 21.742 |  |
|  |  | Std. Deviation | | 4.66281 |  |
|  |  | Minimum | | .00 |  |
|  |  | Maximum | | 18.00 |  |
|  |  | Range | | 18.00 |  |
|  |  | Interquartile Range | | 5.30 |  |
|  |  | Skewness | | .915 | .361 |
|  |  | Kurtosis | | .172 | .709 |
|  | Ya | Mean | | 6.2273 | .95426 |
|  |  | 95% Confidence Interval for Mean | Lower Bound | 4.2428 |  |
|  |  |  | Upper Bound | 8.2118 |  |
|  |  | 5% Trimmed Mean | | 5.9753 |  |
|  |  | Median | | 5.0000 |  |
|  |  | Variance | | 20.034 |  |
|  |  | Std. Deviation | | 4.47588 |  |
|  |  | Minimum | | .30 |  |
|  |  | Maximum | | 17.00 |  |
|  |  | Range | | 16.70 |  |
|  |  | Interquartile Range | | 8.00 |  |
|  |  | Skewness | | .704 | .491 |
|  |  | Kurtosis | | -.161 | .953 |

| **Percentiles** | | | | | | | | | |
| --- | --- | --- | --- | --- | --- | --- | --- | --- | --- |
|  |  | edema | Percentiles | | | | | | |
|  |  |  | 5 | 10 | 25 | 50 | 75 | 90 | 95 |
| Weighted Average(Definition 1) | Usia (tahun) | Tidak | .6000 | 1.1400 | 3.0000 | 5.5000 | 8.3000 | 14.0000 | 17.2800 |
|  |  | Ya | .4050 | 1.1500 | 2.0000 | 5.0000 | 10.0000 | 12.6300 | 16.3850 |
| Tukey's Hinges | Usia (tahun) | Tidak |  |  | 3.5000 | 5.5000 | 8.1500 |  |  |
|  |  | Ya |  |  | 2.0000 | 5.0000 | 10.0000 |  |  |

| **Tests of Normality** | | | | | | | |
| --- | --- | --- | --- | --- | --- | --- | --- |
|  | edema | Kolmogorov-Smirnov^a^ | | | Shapiro-Wilk | | |
|  |  | Statistic | df | Sig. | Statistic | df | Sig. |
| Usia (tahun) | Tidak | .136 | 43 | .043 | .919 | 43 | .005 |
|  | Ya | .153 | 22 | .194 | .932 | 22 | .137 |
| a. Lilliefors Significance Correction | | | | | | | |

**Usia (tahun)**

**NPar Tests**

**Mann-Whitney Test**

| **Ranks** | | | | |
| --- | --- | --- | --- | --- |
|  | edema | N | Mean Rank | Sum of Ranks |
| Usia (tahun) | Tidak | 43 | 33.49 | 1440.00 |
|  | Ya | 22 | 32.05 | 705.00 |
|  | Total | 65 |  |  |

| **Test Statistics^a^** | |
| --- | --- |
|  | Usia (tahun) |
| Mann-Whitney U | 452.000 |
| Wilcoxon W | 705.000 |
| Z | -.292 |
| Asymp. Sig. (2-tailed) | .771 |
| a. Grouping Variable: edema | |

**Explore**

**Perdarahan**

| **Case Processing Summary** | | | | | | | |
| --- | --- | --- | --- | --- | --- | --- | --- |
|  | Perdarahan | Cases | | | | | |
|  |  | Valid | | Missing | | Total | |
|  |  | N | Percent | N | Percent | N | Percent |
| Usia (tahun) | Tidak | 58 | 100.0% | 0 | 0.0% | 58 | 100.0% |
|  | Ya | 7 | 100.0% | 0 | 0.0% | 7 | 100.0% |

| **Descriptives** | | | | | |
| --- | --- | --- | --- | --- | --- |
|  | Perdarahan | | | Statistic | Std. Error |
| Usia (tahun) | Tidak | Mean | | 6.2905 | .59625 |
|  |  | 95% Confidence Interval for Mean | Lower Bound | 5.0965 |  |
|  |  |  | Upper Bound | 7.4845 |  |
|  |  | 5% Trimmed Mean | | 6.0172 |  |
|  |  | Median | | 5.0000 |  |
|  |  | Variance | | 20.620 |  |
|  |  | Std. Deviation | | 4.54090 |  |
|  |  | Minimum | | .00 |  |
|  |  | Maximum | | 18.00 |  |
|  |  | Range | | 18.00 |  |
|  |  | Interquartile Range | | 7.25 |  |
|  |  | Skewness | | .860 | .314 |
|  |  | Kurtosis | | .016 | .618 |
|  | Ya | Mean | | 7.7143 | 1.87355 |
|  |  | 95% Confidence Interval for Mean | Lower Bound | 3.1299 |  |
|  |  |  | Upper Bound | 12.2987 |  |
|  |  | 5% Trimmed Mean | | 7.5714 |  |
|  |  | Median | | 7.0000 |  |
|  |  | Variance | | 24.571 |  |
|  |  | Std. Deviation | | 4.95696 |  |
|  |  | Minimum | | 1.00 |  |
|  |  | Maximum | | 17.00 |  |
|  |  | Range | | 16.00 |  |
|  |  | Interquartile Range | | 5.00 |  |
|  |  | Skewness | | .928 | .794 |
|  |  | Kurtosis | | 1.987 | 1.587 |

| **Percentiles** | | | | | | | | | |
| --- | --- | --- | --- | --- | --- | --- | --- | --- | --- |
|  |  | Perdarahan | Percentiles | | | | | | |
|  |  |  | 5 | 10 | 25 | 50 | 75 | 90 | 95 |
| Weighted Average(Definition 1) | Usia (tahun) | Tidak | .4900 | 1.1900 | 2.7500 | 5.0000 | 10.0000 | 13.5500 | 16.0800 |
|  |  | Ya | 1.0000 | 1.0000 | 5.0000 | 7.0000 | 10.0000 | . | . |
| Tukey's Hinges | Usia (tahun) | Tidak |  |  | 2.8000 | 5.0000 | 10.0000 |  |  |
|  |  | Ya |  |  | 5.5000 | 7.0000 | 9.0000 |  |  |

| **Tests of Normality** | | | | | | | |
| --- | --- | --- | --- | --- | --- | --- | --- |
|  | Perdarahan | Kolmogorov-Smirnov^a^ | | | Shapiro-Wilk | | |
|  |  | Statistic | df | Sig. | Statistic | df | Sig. |
| Usia (tahun) | Tidak | .138 | 58 | .008 | .923 | 58 | .001 |
|  | Ya | .191 | 7 | .200^*^ | .939 | 7 | .631 |
| *. This is a lower bound of the true significance. | | | | | | | |
| a. Lilliefors Significance Correction | | | | | | | |

**Usia (tahun)**

**NPar Tests**

**Mann-Whitney Test**

| **Ranks** | | | | |
| --- | --- | --- | --- | --- |
|  | Perdarahan | N | Mean Rank | Sum of Ranks |
| Usia (tahun) | Tidak | 58 | 32.24 | 1870.00 |
|  | Ya | 7 | 39.29 | 275.00 |
|  | Total | 65 |  |  |

| **Test Statistics^a^** | |
| --- | --- |
|  | Usia (tahun) |
| Mann-Whitney U | 159.000 |
| Wilcoxon W | 1870.000 |
| Z | -.933 |
| Asymp. Sig. (2-tailed) | .351 |
| a. Grouping Variable: Perdarahan | |

**Explore**

**Pus**

| **Case Processing Summary** | | | | | | | |
| --- | --- | --- | --- | --- | --- | --- | --- |
|  | Pus | Cases | | | | | |
|  |  | Valid | | Missing | | Total | |
|  |  | N | Percent | N | Percent | N | Percent |
| Usia (tahun) | Tidak | 58 | 100.0% | 0 | 0.0% | 58 | 100.0% |
|  | Ya | 7 | 100.0% | 0 | 0.0% | 7 | 100.0% |

| **Descriptives** | | | | | |
| --- | --- | --- | --- | --- | --- |
|  | Pus | | | Statistic | Std. Error |
| Usia (tahun) | Tidak | Mean | | 5.8353 | .54467 |
|  |  | 95% Confidence Interval for Mean | Lower Bound | 4.7447 |  |
|  |  |  | Upper Bound | 6.9260 |  |
|  |  | 5% Trimmed Mean | | 5.5115 |  |
|  |  | Median | | 5.0000 |  |
|  |  | Variance | | 17.207 |  |
|  |  | Std. Deviation | | 4.14811 |  |
|  |  | Minimum | | .00 |  |
|  |  | Maximum | | 18.00 |  |
|  |  | Range | | 18.00 |  |
|  |  | Interquartile Range | | 5.25 |  |
|  |  | Skewness | | 1.138 | .314 |
|  |  | Kurtosis | | 1.253 | .618 |
|  | Ya | Mean | | 11.4857 | 1.92235 |
|  |  | 95% Confidence Interval for Mean | Lower Bound | 6.7819 |  |
|  |  |  | Upper Bound | 16.1895 |  |
|  |  | 5% Trimmed Mean | | 11.7619 |  |
|  |  | Median | | 12.9000 |  |
|  |  | Variance | | 25.868 |  |
|  |  | Std. Deviation | | 5.08607 |  |
|  |  | Minimum | | 1.00 |  |
|  |  | Maximum | | 17.00 |  |
|  |  | Range | | 16.00 |  |
|  |  | Interquartile Range | | 4.00 |  |
|  |  | Skewness | | -1.705 | .794 |
|  |  | Kurtosis | | 3.721 | 1.587 |

| **Percentiles** | | | | | | | | | |
| --- | --- | --- | --- | --- | --- | --- | --- | --- | --- |
|  |  | Pus | Percentiles | | | | | | |
|  |  |  | 5 | 10 | 25 | 50 | 75 | 90 | 95 |
| Weighted Average(Definition 1) | Usia (tahun) | Tidak | .4900 | 1.1900 | 2.7500 | 5.0000 | 8.0000 | 11.1600 | 16.0800 |
|  |  | Ya | 1.0000 | 1.0000 | 10.0000 | 12.9000 | 14.0000 | . | . |
| Tukey's Hinges | Usia (tahun) | Tidak |  |  | 2.8000 | 5.0000 | 8.0000 |  |  |
|  |  | Ya |  |  | 11.0000 | 12.9000 | 13.7500 |  |  |

| **Tests of Normality** | | | | | | | |
| --- | --- | --- | --- | --- | --- | --- | --- |
|  | Pus | Kolmogorov-Smirnov^a^ | | | Shapiro-Wilk | | |
|  |  | Statistic | df | Sig. | Statistic | df | Sig. |
| Usia (tahun) | Tidak | .122 | 58 | .031 | .911 | 58 | .000 |
|  | Ya | .255 | 7 | .189 | .836 | 7 | .092 |
| a. Lilliefors Significance Correction | | | | | | | |

**Usia (tahun)**

**NPar Tests**

**Mann-Whitney Test**

| **Ranks** | | | | |
| --- | --- | --- | --- | --- |
|  | Pus | N | Mean Rank | Sum of Ranks |
| Usia (tahun) | Tidak | 58 | 30.91 | 1792.50 |
|  | Ya | 7 | 50.36 | 352.50 |
|  | Total | 65 |  |  |

| **Test Statistics^a^** | |
| --- | --- |
|  | Usia (tahun) |
| Mann-Whitney U | 81.500 |
| Wilcoxon W | 1792.500 |
| Z | -2.575 |
| Asymp. Sig. (2-tailed) | .010 |
| a. Grouping Variable: Pus | |

**Explore**

**Dehisens**

| **Case Processing Summary** | | | | | | | |
| --- | --- | --- | --- | --- | --- | --- | --- |
|  | Dehisens | Cases | | | | | |
|  |  | Valid | | Missing | | Total | |
|  |  | N | Percent | N | Percent | N | Percent |
| Usia (tahun) | Tidak | 59 | 100.0% | 0 | 0.0% | 59 | 100.0% |
|  | Ya | 6 | 100.0% | 0 | 0.0% | 6 | 100.0% |

| **Descriptives** | | | | | |
| --- | --- | --- | --- | --- | --- |
|  | Dehisens | | | Statistic | Std. Error |
| Usia (tahun) | Tidak | Mean | | 6.2093 | .57220 |
|  |  | 95% Confidence Interval for Mean | Lower Bound | 5.0639 |  |
|  |  |  | Upper Bound | 7.3547 |  |
|  |  | 5% Trimmed Mean | | 5.9280 |  |
|  |  | Median | | 5.0000 |  |
|  |  | Variance | | 19.317 |  |
|  |  | Std. Deviation | | 4.39511 |  |
|  |  | Minimum | | .00 |  |
|  |  | Maximum | | 18.00 |  |
|  |  | Range | | 18.00 |  |
|  |  | Interquartile Range | | 5.10 |  |
|  |  | Skewness | | .948 | .311 |
|  |  | Kurtosis | | .399 | .613 |
|  | Ya | Mean | | 8.7500 | 2.44864 |
|  |  | 95% Confidence Interval for Mean | Lower Bound | 2.4556 |  |
|  |  |  | Upper Bound | 15.0444 |  |
|  |  | 5% Trimmed Mean | | 8.6944 |  |
|  |  | Median | | 10.0000 |  |
|  |  | Variance | | 35.975 |  |
|  |  | Std. Deviation | | 5.99792 |  |
|  |  | Minimum | | 1.50 |  |
|  |  | Maximum | | 17.00 |  |
|  |  | Range | | 15.50 |  |
|  |  | Interquartile Range | | 11.38 |  |
|  |  | Skewness | | -.124 | .845 |
|  |  | Kurtosis | | -1.064 | 1.741 |

| **Percentiles** | | | | | | | | | |
| --- | --- | --- | --- | --- | --- | --- | --- | --- | --- |
|  |  | Dehisens | Percentiles | | | | | | |
|  |  |  | 5 | 10 | 25 | 50 | 75 | 90 | 95 |
| Weighted Average(Definition 1) | Usia (tahun) | Tidak | .5000 | 1.1000 | 2.9000 | 5.0000 | 8.0000 | 13.5000 | 16.0000 |
|  |  | Ya | 1.5000 | 1.5000 | 1.8750 | 10.0000 | 13.2500 | . | . |
| Tukey's Hinges | Usia (tahun) | Tidak |  |  | 2.9500 | 5.0000 | 8.0000 |  |  |
|  |  | Ya |  |  | 2.0000 | 10.0000 | 12.0000 |  |  |

| **Tests of Normality** | | | | | | | |
| --- | --- | --- | --- | --- | --- | --- | --- |
|  | Dehisens | Kolmogorov-Smirnov^a^ | | | Shapiro-Wilk | | |
|  |  | Statistic | df | Sig. | Statistic | df | Sig. |
| Usia (tahun) | Tidak | .129 | 59 | .016 | .922 | 59 | .001 |
|  | Ya | .249 | 6 | .200^*^ | .907 | 6 | .417 |
| *. This is a lower bound of the true significance. | | | | | | | |
| a. Lilliefors Significance Correction | | | | | | | |

**Usia (tahun)**

**NPar Tests**

**Mann-Whitney Test**

| **Ranks** | | | | |
| --- | --- | --- | --- | --- |
|  | Dehisens | N | Mean Rank | Sum of Ranks |
| Usia (tahun) | Tidak | 59 | 32.26 | 1903.50 |
|  | Ya | 6 | 40.25 | 241.50 |
|  | Total | 65 |  |  |

| **Test Statistics^a^** | |
| --- | --- |
|  | Usia (tahun) |
| Mann-Whitney U | 133.500 |
| Wilcoxon W | 1903.500 |
| Z | -.987 |
| Asymp. Sig. (2-tailed) | .323 |
| Exact Sig. [2*(1-tailed Sig.)] | .333^b^ |
| a. Grouping Variable: Dehisens | |
| b. Not corrected for ties. | |

**Explore**

**Fistula**

| **Case Processing Summary** | | | | | | | |
| --- | --- | --- | --- | --- | --- | --- | --- |
|  | Fistula | Cases | | | | | |
|  |  | Valid | | Missing | | Total | |
|  |  | N | Percent | N | Percent | N | Percent |
| Usia (tahun) | Tidak | 60 | 100.0% | 0 | 0.0% | 60 | 100.0% |
|  | Ya | 5 | 100.0% | 0 | 0.0% | 5 | 100.0% |

| **Descriptives** | | | | | |
| --- | --- | --- | --- | --- | --- |
|  | Fistula | | | Statistic | Std. Error |
| Usia (tahun) | Tidak | Mean | | 6.4342 | .57199 |
|  |  | 95% Confidence Interval for Mean | Lower Bound | 5.2896 |  |
|  |  |  | Upper Bound | 7.5787 |  |
|  |  | 5% Trimmed Mean | | 6.1972 |  |
|  |  | Median | | 5.3750 |  |
|  |  | Variance | | 19.630 |  |
|  |  | Std. Deviation | | 4.43058 |  |
|  |  | Minimum | | .00 |  |
|  |  | Maximum | | 17.60 |  |
|  |  | Range | | 17.60 |  |
|  |  | Interquartile Range | | 7.08 |  |
|  |  | Skewness | | .731 | .309 |
|  |  | Kurtosis | | -.150 | .608 |
|  | Ya | Mean | | 6.5600 | 2.97634 |
|  |  | 95% Confidence Interval for Mean | Lower Bound | -1.7036 |  |
|  |  |  | Upper Bound | 14.8236 |  |
|  |  | 5% Trimmed Mean | | 6.2056 |  |
|  |  | Median | | 4.0000 |  |
|  |  | Variance | | 44.293 |  |
|  |  | Std. Deviation | | 6.65530 |  |
|  |  | Minimum | | 1.50 |  |
|  |  | Maximum | | 18.00 |  |
|  |  | Range | | 16.50 |  |
|  |  | Interquartile Range | | 10.10 |  |
|  |  | Skewness | | 1.834 | .913 |
|  |  | Kurtosis | | 3.485 | 2.000 |

| **Percentiles** | | | | | | | | | |
| --- | --- | --- | --- | --- | --- | --- | --- | --- | --- |
|  |  | Fistula | Percentiles | | | | | | |
|  |  |  | 5 | 10 | 25 | 50 | 75 | 90 | 95 |
| Weighted Average(Definition 1) | Usia (tahun) | Tidak | .5250 | 1.1100 | 2.9250 | 5.3750 | 10.0000 | 13.4400 | 15.9000 |
|  |  | Ya | 1.5000 | 1.5000 | 2.1500 | 4.0000 | 12.2500 | . | . |
| Tukey's Hinges | Usia (tahun) | Tidak |  |  | 2.9500 | 5.3750 | 10.0000 |  |  |
|  |  | Ya |  |  | 2.8000 | 4.0000 | 6.5000 |  |  |

| **Tests of Normality** | | | | | | | |
| --- | --- | --- | --- | --- | --- | --- | --- |
|  | Fistula | Kolmogorov-Smirnov^a^ | | | Shapiro-Wilk | | |
|  |  | Statistic | df | Sig. | Statistic | df | Sig. |
| Usia (tahun) | Tidak | .122 | 60 | .026 | .940 | 60 | .005 |
|  | Ya | .304 | 5 | .148 | .794 | 5 | .072 |
| a. Lilliefors Significance Correction | | | | | | | |

**Usia (tahun)**

**NPar Tests**

**Mann-Whitney Test**

| **Ranks** | | | | |
| --- | --- | --- | --- | --- |
|  | Fistula | N | Mean Rank | Sum of Ranks |
| Usia (tahun) | Tidak | 60 | 33.25 | 1995.00 |
|  | Ya | 5 | 30.00 | 150.00 |
|  | Total | 65 |  |  |

| **Test Statistics^a^** | |
| --- | --- |
|  | Usia (tahun) |
| Mann-Whitney U | 135.000 |
| Wilcoxon W | 150.000 |
| Z | -.370 |
| Asymp. Sig. (2-tailed) | .711 |
| Exact Sig. [2*(1-tailed Sig.)] | .729^b^ |
| a. Grouping Variable: Fistula | |
| b. Not corrected for ties. | |

**Explore**

**Kultur Positif**

| **Case Processing Summary** | | | | | | | |
| --- | --- | --- | --- | --- | --- | --- | --- |
|  | Kultur Positif | Cases | | | | | |
|  |  | Valid | | Missing | | Total | |
|  |  | N | Percent | N | Percent | N | Percent |
| Usia (tahun) | Tidak | 37 | 100.0% | 0 | 0.0% | 37 | 100.0% |
|  | Ya | 28 | 100.0% | 0 | 0.0% | 28 | 100.0% |

| **Descriptives** | | | | | |
| --- | --- | --- | --- | --- | --- |
|  | Kultur Positif | | | Statistic | Std. Error |
| Usia (tahun) | Tidak | Mean | | 6.4230 | .73083 |
|  |  | 95% Confidence Interval for Mean | Lower Bound | 4.9408 |  |
|  |  |  | Upper Bound | 7.9052 |  |
|  |  | 5% Trimmed Mean | | 6.1545 |  |
|  |  | Median | | 5.0000 |  |
|  |  | Variance | | 19.762 |  |
|  |  | Std. Deviation | | 4.44548 |  |
|  |  | Minimum | | .00 |  |
|  |  | Maximum | | 18.00 |  |
|  |  | Range | | 18.00 |  |
|  |  | Interquartile Range | | 4.00 |  |
|  |  | Skewness | | 1.130 | .388 |
|  |  | Kurtosis | | .891 | .759 |
|  | Ya | Mean | | 6.4714 | .90847 |
|  |  | 95% Confidence Interval for Mean | Lower Bound | 4.6074 |  |
|  |  |  | Upper Bound | 8.3355 |  |
|  |  | 5% Trimmed Mean | | 6.2341 |  |
|  |  | Median | | 5.5500 |  |
|  |  | Variance | | 23.109 |  |
|  |  | Std. Deviation | | 4.80716 |  |
|  |  | Minimum | | .50 |  |
|  |  | Maximum | | 17.60 |  |
|  |  | Range | | 17.10 |  |
|  |  | Interquartile Range | | 8.00 |  |
|  |  | Skewness | | .561 | .441 |
|  |  | Kurtosis | | -.686 | .858 |

| **Percentiles** | | | | | | | | | |
| --- | --- | --- | --- | --- | --- | --- | --- | --- | --- |
|  |  | Kultur Positif | Percentiles | | | | | | |
|  |  |  | 5 | 10 | 25 | 50 | 75 | 90 | 95 |
| Weighted Average(Definition 1) | Usia (tahun) | Tidak | .2700 | 1.9000 | 4.0000 | 5.0000 | 8.0000 | 14.4000 | 17.1000 |
|  |  | Ya | .7250 | 1.0000 | 2.0000 | 5.5500 | 10.0000 | 13.5500 | 15.9800 |
| Tukey's Hinges | Usia (tahun) | Tidak |  |  | 4.0000 | 5.0000 | 8.0000 |  |  |
|  |  | Ya |  |  | 2.0000 | 5.5500 | 10.0000 |  |  |

| **Tests of Normality** | | | | | | | |
| --- | --- | --- | --- | --- | --- | --- | --- |
|  | Kultur Positif | Kolmogorov-Smirnov^a^ | | | Shapiro-Wilk | | |
|  |  | Statistic | df | Sig. | Statistic | df | Sig. |
| Usia (tahun) | Tidak | .160 | 37 | .018 | .895 | 37 | .002 |
|  | Ya | .158 | 28 | .072 | .926 | 28 | .048 |
| a. Lilliefors Significance Correction | | | | | | | |

**Usia (tahun)**

**NPar Tests**

**Mann-Whitney Test**

| **Ranks** | | | | |
| --- | --- | --- | --- | --- |
|  | Kultur Positif | N | Mean Rank | Sum of Ranks |
| Usia (tahun) | Tidak | 37 | 33.12 | 1225.50 |
|  | Ya | 28 | 32.84 | 919.50 |
|  | Total | 65 |  |  |

| **Test Statistics^a^** | |
| --- | --- |
|  | Usia (tahun) |
| Mann-Whitney U | 513.500 |
| Wilcoxon W | 919.500 |
| Z | -.060 |
| Asymp. Sig. (2-tailed) | .952 |
| a. Grouping Variable: Kultur Positif | |

**Explore**

**Positivitas**

| **Case Processing Summary** | | | | | | | |
| --- | --- | --- | --- | --- | --- | --- | --- |
|  | Positivitas | Cases | | | | | |
|  |  | Valid | | Missing | | Total | |
|  |  | N | Percent | N | Percent | N | Percent |
| Usia (tahun) | Klinis -, Kultur - | 33 | 100.0% | 0 | 0.0% | 33 | 100.0% |
|  | Klinis +, Kultur + | 28 | 100.0% | 0 | 0.0% | 28 | 100.0% |
|  | Klinis +, Kultur - | 4 | 100.0% | 0 | 0.0% | 4 | 100.0% |

| **Descriptives** | | | | | |
| --- | --- | --- | --- | --- | --- |
|  | Positivitas | | | Statistic | Std. Error |
| Usia (tahun) | Klinis -, Kultur - | Mean | | 6.2773 | .71660 |
|  |  | 95% Confidence Interval for Mean | Lower Bound | 4.8176 |  |
|  |  |  | Upper Bound | 7.7369 |  |
|  |  | 5% Trimmed Mean | | 6.0120 |  |
|  |  | Median | | 5.0000 |  |
|  |  | Variance | | 16.946 |  |
|  |  | Std. Deviation | | 4.11657 |  |
|  |  | Minimum | | .00 |  |
|  |  | Maximum | | 18.00 |  |
|  |  | Range | | 18.00 |  |
|  |  | Interquartile Range | | 3.95 |  |
|  |  | Skewness | | 1.238 | .409 |
|  |  | Kurtosis | | 1.591 | .798 |
|  | Klinis +, Kultur + | Mean | | 6.4714 | .90847 |
|  |  | 95% Confidence Interval for Mean | Lower Bound | 4.6074 |  |
|  |  |  | Upper Bound | 8.3355 |  |
|  |  | 5% Trimmed Mean | | 6.2341 |  |
|  |  | Median | | 5.5500 |  |
|  |  | Variance | | 23.109 |  |
|  |  | Std. Deviation | | 4.80716 |  |
|  |  | Minimum | | .50 |  |
|  |  | Maximum | | 17.60 |  |
|  |  | Range | | 17.10 |  |
|  |  | Interquartile Range | | 8.00 |  |
|  |  | Skewness | | .561 | .441 |
|  |  | Kurtosis | | -.686 | .858 |
|  | Klinis +, Kultur - | Mean | | 7.6250 | 3.68202 |
|  |  | 95% Confidence Interval for Mean | Lower Bound | -4.0928 |  |
|  |  |  | Upper Bound | 19.3428 |  |
|  |  | 5% Trimmed Mean | | 7.4444 |  |
|  |  | Median | | 6.0000 |  |
|  |  | Variance | | 54.229 |  |
|  |  | Std. Deviation | | 7.36405 |  |
|  |  | Minimum | | 1.50 |  |
|  |  | Maximum | | 17.00 |  |
|  |  | Range | | 15.50 |  |
|  |  | Interquartile Range | | 13.63 |  |
|  |  | Skewness | | .717 | 1.014 |
|  |  | Kurtosis | | -1.978 | 2.619 |

| **Percentiles** | | | | | | | | | | |
| --- | --- | --- | --- | --- | --- | --- | --- | --- | --- | --- |
|  |  | Positivitas | Percentiles | | | | | | |  |
|  |  |  | 5 | 10 | 25 | 50 | 75 | 90 | 95 |  |
| Weighted Average(Definition 1) | Usia (tahun) | Klinis -, Kultur - | .2100 | 2.0000 | 4.0000 | 5.0000 | 7.9500 | 13.4400 | 16.6000 |  |
|  |  | Klinis +, Kultur + | .7250 | 1.0000 | 2.0000 | 5.5500 | 10.0000 | 13.5500 | 15.9800 |  |
|  |  | Klinis +, Kultur - | 1.5000 | 1.5000 | 1.6250 | 6.0000 | 15.2500 | . | . |  |
| Tukey's Hinges | Usia (tahun) | Klinis -, Kultur - |  |  | 4.0000 | 5.0000 | 7.9000 |  |  |  |
|  |  | Klinis +, Kultur + |  |  | 2.0000 | 5.5500 | 10.0000 |  |  |  |
|  |  | Klinis +, Kultur - |  |  | 1.7500 | 6.0000 | 13.5000 |  |  |  |

| **Tests of Normality** | | | | | | | |
| --- | --- | --- | --- | --- | --- | --- | --- |
|  | Positivitas | Kolmogorov-Smirnov^a^ | | | Shapiro-Wilk | | |
|  |  | Statistic | df | Sig. | Statistic | df | Sig. |
| Usia (tahun) | Klinis -, Kultur - | .163 | 33 | .026 | .891 | 33 | .003 |
|  | Klinis +, Kultur + | .158 | 28 | .072 | .926 | 28 | .048 |
|  | Klinis +, Kultur - | .278 | 4 | . | .883 | 4 | .350 |
| a. Lilliefors Significance Correction | | | | | | | |

**Usia (tahun)**

**Nonparametric Tests**

| **Hypothesis Test Summary** | | | | |
| --- | --- | --- | --- | --- |
|  | Null Hypothesis | Test | Sig. | Decision |
| 1 | The distribution of Usia (tahun) is the same across categories of Positivitas. | Independent-Samples Kruskal-Wallis Test | .997 | Retain the null hypothesis. |
| Asymptotic significances are displayed. The significance level is .050. | | | | |

**Independent-Samples Kruskal-Wallis Test**

**Usia (tahun) across Positivitas**

| **Independent-Samples Kruskal-Wallis Test Summary** | |
| --- | --- |
| Total N | 65 |
| Test Statistic | .005^a,b^ |
| Degree Of Freedom | 2 |
| Asymptotic Sig.(2-sided test) | .997 |
| a. The test statistic is adjusted for ties. | |
| b. Multiple comparisons are not performed because the overall test does not show significant differences across samples. | |

**Explore**

**Derajat IDO**

| **Case Processing Summary** | | | | | | | |
| --- | --- | --- | --- | --- | --- | --- | --- |
|  | Derajat IDO | Cases | | | | | |
|  |  | Valid | | Missing | | Total | |
|  |  | N | Percent | N | Percent | N | Percent |
| Usia (tahun) | Tidak | 33 | 100.0% | 0 | 0.0% | 33 | 100.0% |
|  | Dalam | 6 | 100.0% | 0 | 0.0% | 6 | 100.0% |
|  | Superfisial | 26 | 100.0% | 0 | 0.0% | 26 | 100.0% |

| **Descriptives** | | | | | |
| --- | --- | --- | --- | --- | --- |
|  | Derajat IDO | | | Statistic | Std. Error |
| Usia (tahun) | Tidak | Mean | | 6.2773 | .71660 |
|  |  | 95% Confidence Interval for Mean | Lower Bound | 4.8176 |  |
|  |  |  | Upper Bound | 7.7369 |  |
|  |  | 5% Trimmed Mean | | 6.0120 |  |
|  |  | Median | | 5.0000 |  |
|  |  | Variance | | 16.946 |  |
|  |  | Std. Deviation | | 4.11657 |  |
|  |  | Minimum | | .00 |  |
|  |  | Maximum | | 18.00 |  |
|  |  | Range | | 18.00 |  |
|  |  | Interquartile Range | | 3.95 |  |
|  |  | Skewness | | 1.238 | .409 |
|  |  | Kurtosis | | 1.591 | .798 |
|  | Dalam | Mean | | 8.7500 | 2.44864 |
|  |  | 95% Confidence Interval for Mean | Lower Bound | 2.4556 |  |
|  |  |  | Upper Bound | 15.0444 |  |
|  |  | 5% Trimmed Mean | | 8.6944 |  |
|  |  | Median | | 10.0000 |  |
|  |  | Variance | | 35.975 |  |
|  |  | Std. Deviation | | 5.99792 |  |
|  |  | Minimum | | 1.50 |  |
|  |  | Maximum | | 17.00 |  |
|  |  | Range | | 15.50 |  |
|  |  | Interquartile Range | | 11.38 |  |
|  |  | Skewness | | -.124 | .845 |
|  |  | Kurtosis | | -1.064 | 1.741 |
|  | Superfisial | Mean | | 6.1231 | .94280 |
|  |  | 95% Confidence Interval for Mean | Lower Bound | 4.1813 |  |
|  |  |  | Upper Bound | 8.0648 |  |
|  |  | 5% Trimmed Mean | | 5.8376 |  |
|  |  | Median | | 5.2500 |  |
|  |  | Variance | | 23.111 |  |
|  |  | Std. Deviation | | 4.80735 |  |
|  |  | Minimum | | .50 |  |
|  |  | Maximum | | 17.60 |  |
|  |  | Range | | 17.10 |  |
|  |  | Interquartile Range | | 8.00 |  |
|  |  | Skewness | | .762 | .456 |
|  |  | Kurtosis | | -.337 | .887 |

| **Percentiles** | | | | | | | | | |
| --- | --- | --- | --- | --- | --- | --- | --- | --- | --- |
|  |  | Derajat IDO | Percentiles | | | | | | |
|  |  |  | 5 | 10 | 25 | 50 | 75 | 90 | 95 |
| Weighted Average(Definition 1) | Usia (tahun) | Tidak | .2100 | 2.0000 | 4.0000 | 5.0000 | 7.9500 | 13.4400 | 16.6000 |
|  |  | Dalam | 1.5000 | 1.5000 | 1.8750 | 10.0000 | 13.2500 | . | . |
|  |  | Superfisial | .6750 | 1.0000 | 2.0000 | 5.2500 | 10.0000 | 13.6500 | 16.3400 |
| Tukey's Hinges | Usia (tahun) | Tidak |  |  | 4.0000 | 5.0000 | 7.9000 |  |  |
|  |  | Dalam |  |  | 2.0000 | 10.0000 | 12.0000 |  |  |
|  |  | Superfisial |  |  | 2.0000 | 5.2500 | 10.0000 |  |  |

| **Tests of Normality** | | | | | | | |
| --- | --- | --- | --- | --- | --- | --- | --- |
|  | Derajat IDO | Kolmogorov-Smirnov^a^ | | | Shapiro-Wilk | | |
|  |  | Statistic | df | Sig. | Statistic | df | Sig. |
| Usia (tahun) | Tidak | .163 | 33 | .026 | .891 | 33 | .003 |
|  | Dalam | .249 | 6 | .200^*^ | .907 | 6 | .417 |
|  | Superfisial | .165 | 26 | .066 | .911 | 26 | .029 |
| *. This is a lower bound of the true significance. | | | | | | | |
| a. Lilliefors Significance Correction | | | | | | | |

**Nonparametric Tests**

**Independent-Samples Kruskal-Wallis Test**

**Usia (tahun) across Derajat IDO**

| **Independent-Samples Kruskal-Wallis Test Summary** | |
| --- | --- |
| Total N | 65 |
| Test Statistic | 1.114^a,b^ |
| Degree Of Freedom | 2 |
| Asymptotic Sig.(2-sided test) | .573 |
| a. The test statistic is adjusted for ties. | |
| b. Multiple comparisons are not performed because the overall test does not show significant differences across samples. | |
